# Hippocampal Somatostatin Interneurons, Long-Term Synaptic Plasticity and Memory

**DOI:** 10.3389/fncir.2021.687558

**Published:** 2021-06-02

**Authors:** Eve Honoré, Abdessattar Khlaifia, Anthony Bosson, Jean-Claude Lacaille

**Affiliations:** Department of Neurosciences, Centre for Interdisciplinary Research on Brain and Learning, Research Group on the Central Nervous System, Université de Montréal, Montreal, QC, Canada

**Keywords:** somatostatin, inhibitory interneuron, hippocampus, network metaplasticity, long-term potentiation, spatial and contextual memory, memory impairment, Alzheimer’s disease

## Abstract

A distinctive feature of the hippocampal structure is the diversity of inhibitory interneurons. These complex inhibitory interconnections largely contribute to the tight modulation of hippocampal circuitry, as well as to the formation and coordination of neuronal assemblies underlying learning and memory. Inhibitory interneurons provide more than a simple transitory inhibition of hippocampal principal cells (PCs). The synaptic plasticity of inhibitory neurons provides long-lasting changes in the hippocampal network and is a key component of memory formation. The dendrite targeting interneurons expressing the peptide somatostatin (SOM) are particularly interesting in this regard because they display unique long-lasting synaptic changes leading to metaplastic regulation of hippocampal networks. In this article, we examine the actions of the neuropeptide SOM on hippocampal cells, synaptic plasticity, learning, and memory. We address the different subtypes of hippocampal SOM interneurons. We describe the long-term synaptic plasticity that takes place at the excitatory synapses of SOM interneurons, its singular induction and expression mechanisms, as well as the consequences of these changes on the hippocampal network, learning, and memory. We also review evidence that astrocytes provide cell-specific dynamic regulation of inhibition of PC dendrites by SOM interneurons. Finally, we cover how, in mouse models of Alzheimer’s disease (AD), dysfunction of plasticity of SOM interneuron excitatory synapses may also contribute to cognitive impairments in brain disorders.

## Introduction

Hippocampal learning and memory emerge from the proper routing of information throughout its networks and the formation of enduring neuronal assemblies encoding a memory (Kandel et al., 2014). These neuronal assemblies, also called engrams, are formed by a discrete population of excitatory glutamatergic principal cells (PCs) between which synaptic transmission is potentiated (Josselyn and Frankland, 2018; Tonegawa et al., 2018).

A distinctive feature of the hippocampal structure is the diversity of inhibitory interneurons. PCs represent the majority of neurons in every hippocampal region. While interneurons only represent 10 to 20% of the total neuron population, they can be divided into many subgroups based on their laminar position, dendritic and axonal morphology, protein expression, electrophysiological features, and functions (Freund and Buzsáki, 1996; Somogyi and Klausberger, 2005; Tricoire et al., 2011; Kepecs and Fishell, 2014; Pelkey et al., 2017; Booker and Vida, 2018). Interestingly, the different interneuron types have preferred synaptic connections on specific and distinct subcellular domains of the principal cells including the apical and proximal dendrites, cell body, and the axon initial segment. Due to their specific characteristics, inhibitory interneurons play various roles in fine–tuning signal integration and firing of PCs. Additional levels of control of the hippocampal network is achieved by interneurons targeting other interneurons (Katona et al., 1999; Pelkey et al., 2017; Artinian and Lacaille, 2018) along with interneuron-astrocyte interactions (Mederos and Perea, 2019).

These complex inhibitory interconnections largely contribute to the tight modulation of hippocampal circuitry, providing means for the formation and coordination of neuronal assemblies. Hence, hippocampal interneurons also participate in the mechanisms underlying hippocampus-dependent memory. In this review, we aim to shed light on a major subpopulation of GABAergic interneurons specifically inhibiting PC dendrites and characterized by the expression of the peptide somatostatin (SOM). As the morphological and neurochemical profiles of these interneurons have already been reviewed (Pelkey et al., 2017; Booker and Vida, 2018), we will focus on the role of SOM interneuron activity and synaptic plasticity in the regulation of hippocampal networks and memory functions.

## Somatostatin in The Hippocampus

The peptide SOM, also referred as somatotropin release inhibitory factor (SRIF), was originally discovered in the hypothalamus where it acts as a growth hormone inhibitor (Krulich et al., 1968; Brazeau et al., 1973). In the cerebral cortex, SOM is stored in dense-core vesicles in a specific subset GABAergic interneurons and released by intense neuronal activity (Vezzani et al., 1993; Hou and Yu, 2013; Liguz-Lecznar et al., 2016). SOM can bind to five metabotropic receptors (SST_1-5_R) which are coupled to G protein from the Gi/o and Gq/G11 families. Thus, SSTR activation downregulates adenylyl cyclase activity and activates the phosphoinositide 3-kinase and phospholipase Cβ signaling pathways (Liguz-Lecznar et al., 2016; Günther et al., 2018). SST_1-4_R, but not SST_5_R, are present in the hippocampus in PCs and as auto-receptors in SOM interneurons. Although the distribution of SSTRs overlaps within and across hippocampal regions, SSTR subtypes preferentially occupy specific cell compartments. SST_1_R are preferentially located pre-synaptically, SST_2_R and SST_4_R post-synaptically, and SST_3_R extra-synaptically. Yet, which cell type expresses the receptors and whether they are pre or postsynaptic remains unclear (Csaba and Dournaud, 2001; Liguz-Lecznar et al., 2016; Cammalleri et al., 2019).

The systematic distribution of SSTRs suggests a precise regulation of hippocampal networks by SOM. However, the mechanisms by which SOM regulates hippocampal networks remain ambiguous. Reports of membrane and synaptic effects are diverse and sometimes contradictory. On the one hand, SOM has an inhibitory effect on pyramidal neurons exhibiting hyperpolarizing effect, decreasing evoked and spontaneous activity, persistently reducing NMDA and AMPA currents, and reducing spine density (Pittman and Siggins, 1981; Tallent and Siggins, 1997; Hou and Yu, 2013). On the other hand, SOM also produces excitatory effects on pyramidal cells inducing membrane depolarization, as well as increasing spontaneous and evoked excitatory postsynaptic potentials (EPSPs; Olpe et al., 1980; Delfs and Dichter, 1983; Scharfman and Schwartzkroin, 1988, 1989).

Many factors may explain these discrepancies. First, in many studies, the effect of SOM is concentration-dependent, and the number of PCs responding to SOM application with a depolarization follows an inverted U-shaped curve between 100 pM and 1 μM, until it reaches potent toxic concentrations over 10 μM (Olpe et al., 1980; Pittman and Siggins, 1981; Delfs and Dichter, 1983; Scharfman and Schwartzkroin, 1988; Tallent and Siggins, 1997). Second, the location of SOM application is also important. In rabbit hippocampal slices, SOM application on the soma of CA1 PCs induces membrane depolarization often accompanied by action potentials. When applied on the dendrites of PCs SOM produces depolarization or hyperpolarization. Larger SOM applications further away from PCs induce membrane hyperpolarization (Scharfman and Schwartzkroin, 1988). Third, inhibitory interneurons also express SSTRs. SOM application at the soma of interneurons at the border between *stratum pyramidale* and *oriens* produces a depolarization accompanied by an action potential in these cells. When applied at their dendrites, SOM produces either depolarization followed by increased action potentials, or hyperpolarization also followed by increased action potentials, or a combination of depolarization and action potential discharge followed by a hyperpolarization (Scharfman and Schwartzkroin, 1988).

The effect of SOM on interneuron output also appears complex. Blocking SST_1_R resulted in an increase in SOM concentration but had no effect on GABA concentration *in vivo* (De Bundel et al., 2010). This finding may indicate that SOM release from an interneuron can promote auto-inhibition *via* SST_1_R presynaptic activation without interfering with GABA transmission. Bath application of somatostatin did not affect isolated GABAergic inhibitory postsynaptic currents induced by stimulation close to the pyramidal cell layer, nor the density of inhibitory synapses (Tallent and Siggins, 1997; Hou and Yu, 2013). However, earlier research demonstrated persistent reduction or blockade of spontaneous or evoked inhibitory postsynaptic potentials (Pittman and Siggins, 1981; Scharfman and Schwartzkroin, 1988, 1989).

Finally, another level of complexity with SOM effects arises from its long-lasting action. SOM produces long-lasting increases in spontaneous activity and evoked responses of pyramidal cells *via* postsynaptic effects (Delfs and Dichter, 1983; Scharfman and Schwartzkroin, 1988). In addition, transgenic mice with ablation of the SOM gene or mice with pharmacological depletion of SOM by cysteamine treatment, have normal basal transmission but a deficit in long-term potentiation (LTP) in CA1 (Kluge et al., 2008). Taken altogether this *corpus* of data suggests that SOM has a complex function which is dependent on the location and concentration released, the activated receptor subtype, and experimental conditions (for details see Table 1).

**Table 1:** Effects of somatostatin (SOM) on hippocampal pyramidal cell activity.

Reference	Model	Experiment	Results
Hou and Yu (2013)	Rat hippocampal neuronal-glial culture	1 day SOM application 1 μM.	↘ Spine density (excitatory) and density of pre-post synaptic markers. No effect on inhibitory synapses.
Kluge et al. (2008)	Mouse acute slices	SOM-KO mice or cysteamine application.	SOM KO show normal basal synaptic transmission and ↘ LTP in CA1; same results with cysteamine.
Tallent and Siggins (1997)	Rat, acute slices	1 μM SOM superfusion.	SOM persistently ↘ NMDA and AMPA currents in a time and dose-dependent manner. It has no effect on isolated GABAergic currents elicited by pyramidal layer stimulation.
Scharfman and Schwartzkroin (1989)	Rabbit, acute slices	Pressure application of SOM on distinct CA1 pyramidal cell compartment.	Soma application of SOM depolarizes CA1-PCs and persistently ↘ or blocks IPSPs evoked by *oriens* electrical stimulation.
Scharfman and Schwartzkroin (1988)	Rabbit, acute slices	Pressure application of SOM on distinct CA1 pyramidal cell compartment.	SOM application on pyramidal cell soma elicits depolarization often accompanied by action potentials. On dendrites, it produces depolarization or hyperpolarization. Applied in larger quantities further from the cell it hyperpolarizes CA1-PCs. Potentiation with ↗ spontaneous activity and evoked responses due to postsynaptic effects.
Delfs and Dichter (1983)	Rat cortical neurons in culture	100 pM to 100 μM SOM application just over the studied neuron.	Concentration/response curve inverted U- shaped from 100 pM to 1 μM. 30–50% of the neurons respond ↗ with EPSPs and IPSPs amplitude, and ↗ spontaneous action potentials frequency.
Pittman and Siggins (1981)	Rat, acute slices	Application of 0.12 μM to 1.2 μM SOM superfusion on the slice.	Hyperpolarization of PCs and ↘ evoked and spontaneous activity. Slight ↘IPSPs amplitude. Slight↗ EPSPs amplitude evoked by *radiatum* stimulation.
Olpe et al. (1980)	Rat, *in vivo*, anesthetized	Hippocampal 60 s micro-injection of SOM.	↗ Number of neurons responding with excitation, following ↗ SOM concentration. No alteration of GABA release.

↗ = increase; ↘ = decrease.

Interestingly, at the behavioral level, SOM appears important as a neuromodulator of hippocampal function. Genetic ablation of the SOM gene or SOM depletion by cysteamine impairs contextual fear memory and has no effect on cued fear memory, indicating a specific action on hippocampus-dependent memory (Kluge et al., 2008). Moreover, intracerebroventricular injection of SOM, or a non-hydrolyzable SOM analog, before or after learning, increases active and passive avoidance behaviors 24 h after the acquisition and prevents their extinction (Vécsei and Widerlöv, 1988; Vécsei et al., 1989). However, both treatments have no effect on spatial discrimination learning and reversal learning in the T-maze test (Vécsei et al., 1984). Again, SOM concentration is critical, low concentrations increase passive avoidance memory, while 10-fold higher concentrations have the opposite effect (Vécsei et al., 1984, 1989; Vécsei and Widerlöv, 1988; Schettini, 1991). Pre-training intra-hippocampal injection of SOM or a SST_4_R agonist impairs spatial memory in a dose-dependent manner. In addition, the SST_4_R agonist decreases cued memory and enhances the retention of bar pressing tasks. Intriguingly, the administration of SST_1-3_R agonists does not evoke any behavioral change (Gastambide et al., 2008). The timing of SOM treatment is also critical. When injected before the memory acquisition trials in the 8-arm radial maze task, SOM increases acquisition rates. However, when administered before the memory probe test, it impairs performance. Thus, SOM has different effects on acquisition and memory probe tests (Guillou et al., 1993; Lamirault et al., 2001). Finally, it is important to note that hippocampal injections of SOM or agonists of SST_4_R and SST_2_R have anxiolytic and antidepressant-like effects *via* the inhibition of the hypothalamic-pituitary-adrenal axis. Through this mechanism, SOM also regulates emotions and stress responses which strongly modulate learning and memory (Prévôt et al., 2017; for details see Table 2).

**Table 2:** Effects of somatostatin (SOM) on hippocampus-dependent behavior.

Reference	Model	Experiment	Results
Gastambide et al. (2008)	Mouse	Intra-hippocampal injection of SOM or SSTR agonist at different concentrations before spatial and cued versions of Morris water maze and bar pressing conditioning.	SOM and SST_4_R agonist injection ↘ spatial memory dose-dependently. SST_1-3_R agonist does not affect these behavioral tasks. SST_4_R agonist ↘ cued memory but ↗ bar pressing retention.
Kluge et al. (2008)	Mouse	SOM KO mice or cysteamine injection in wild type mice. Contextual fear conditioning and cued fear conditioning.	SOM KO ↘ contextual fear but not cued fear memory. Pre-conditioning cysteamine injection in wild type mice has the same effect, but post-conditioning injection induces unspecific increase of fear response.
Lamirault et al. (2001)	Mouse	Intra-hippocampal injection of SOM or cysteamine prior spatial discrimination eight-arm radial maze task acquisition or test.	SOM injection prior acquisition (0.2 μg/0.2 μl/hippocampus) ↗ acquisition speed but ↘ performances during the test. Injection before the test does not affect memory.
Guillou et al. (1993)	Mouse	Intra hippocampal injection of SOM or cysteamine prior to spatial discrimination eight-arm radial maze task.	SOM injection (0.2 μg/0.2 μl/hippocampus) ↗ acquisition speed, but impairs the ability to change strategies. Cysteamine injection impairs memory.
Vécsei et al. (1989)	Rat	Intra-cerebroventricular SOM or SOM fragments injection in male rats directly after passive avoidance acquisition.	0.6 nM SOM ↗ avoidance latency and 6 nM ↘ it in a passive avoidance task during the test 24 h after. In open field task 6 nM SOM ↘ rearing and 0.6 nM has no effect. Injections above 12 nM are lethal.
Vécsei and Widerlöv (1988)	Rat	Intra-cerebroventricular SOM injection in male rats 30 min before passive avoidance, or active avoidance acquisition.	In passive avoidance: 1 μg of SOM ↗ avoidance latency at 24 h but not 48 h after acquisition. 10 μg ↘ avoidance latency 24 h after acquisition. In active avoidance: 1 μg SOM ↗ learning curve.
Vécsei et al. (1984)	Rat	Intra-cerebroventricular SOM or subcutaneous cysteamine injection in male rats directly after active avoidance and T-maze acquisition.	SOM ↘ extinction of active avoidance but has no effect on T-maze. Cysteamine ↗ extinction in both behavioral tasks. SOM ↗ locomotion in open field 10 min after injection. Cysteamine ↘ locomotion, rearing, and grooming.

↗ = increase; ↘ = decrease.

Thus, at the behavioral level, SOM is required for proper memory formation. The location, timing, and concentration of SOM release by interneurons appears crucial for SOM functional outcomes, perhaps because of the distribution and types of receptors involved. However, a better understanding of how, where and when SOM is released to fulfill its functions requires new precise tools.

## Hippocampal Somatostatin Interneurons

**Figure 1:**
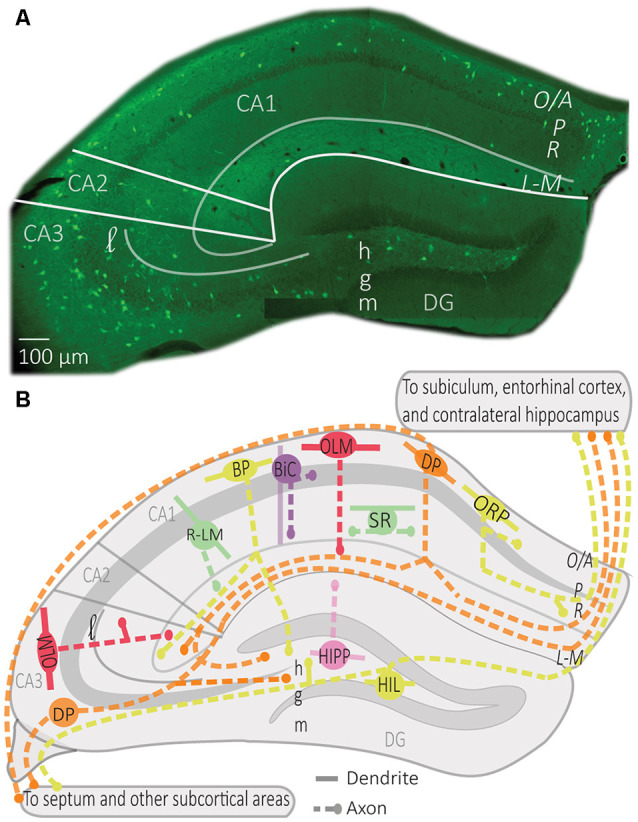
Distribution of somatostatin (SOM) interneurons in hippocampus. **(A)** Representative montage of fluorescence images showing the soma location of SOM interneurons expressing the enhanced yellow fluorescent protein in the dorsal hippocampus of *Sst*^ires-Cre^; *Rosa26*^lsl-EYFP^ mice (Vasuta et al., 2015; Artinian et al., 2019), with indicated anatomical landmarks. **(B)** Simplified diagram illustrating the characteristic position of soma, dendrites, and axons of the different types of SOM interneurons in hippocampus. Abbreviations for hippocampus: CA1–3, *cornu ammonis* 1–3; DG, dentate gyrus; O/A, *oriens*/*alveus*; P, *pyramidale*; R, *radiatum*; L-M, *lacunosum-moleculare*; l, *lucidum*; h, hilus; g, granule cell layer; m, *moleculare*. Abbreviations for SOM interneuron types: OLM, *oriens/lacunosum-moleculare* cell; ORP, *oriens*-retrohippocampal projecting cell; DP, double projecting cell; BP, back-projecting cell; BiC, bistratified cell; R-LM, *radiatum/lacunosum-moleculare* cell; SR, *stratum radiatum* cell; HIPP, hilar perforant path cell; HIL, hilar cell.

Although hippocampal interneurons release the peptide SOM, in hippocampal research this peptide has largely been regarded as a neurochemical marker for a subset of GABAergic interneurons. Through their GABAergic actions, SOM interneurons provide local inhibition to regulate hippocampal networks, and distal inhibition to synchronize hippocampal activity with other brain areas. Hippocampal SOM interneurons have been the subject of recent comprehensive reviews (Muller and Remy, 2014; Pelkey et al., 2017; Booker and Vida, 2018). Thus, they gate the synaptic inputs of their targets (Katona et al., 1999; Muller and Remy, 2014). SOM interneurons preferentially receive excitatory inputs from local PCs to which they send inhibitory feedback (Lacaille et al., 1987; Blasco-Ibáñez and Freund, 1995). Yet, there is more to SOM interneuron function than negative feedback. SOM interneurons dynamically regulate the input-output transformation and firing of PCs both in slices and during exploration *in vivo* (Lovett-Barron et al., 2012; Royer et al., 2012). In addition, SOM interneurons are diverse, and each type has a different contribution to the regulation of information flow through PCs (Figure 1).

### Oriens Lacunosum-Moleculare (OLM) Cells

OLM cells are the most extensively studied SOM interneurons in the hippocampus. They are located in CA3 and CA1 along the complete dorso-ventral axis of the hippocampus (Mikulovic et al., 2015). Their designation comes from the location of their soma and dendrites in *stratum* *oriens* and their rich axonal arborization in *stratum* *lacunosum-moleculare* (Lacaille et al., 1987; Chittajallu et al., 2013; Figure 1). Their main excitatory input comes from local PCs to which they send inhibitory feedback (Lacaille et al., 1987), as well as from cholinergic afferents from the septum and diagonal band of Broca (Lovett-Barron et al., 2012; Sun et al., 2014). They receive inhibition from local inhibitory neurons, mostly vasoactive intestinal peptide (VIP) expressing interneurons, and inhibitory afferents from septal regions (Tyan et al., 2014) and the nucleus incertus (Szonyi et al., 2019).

**Figure 2:**
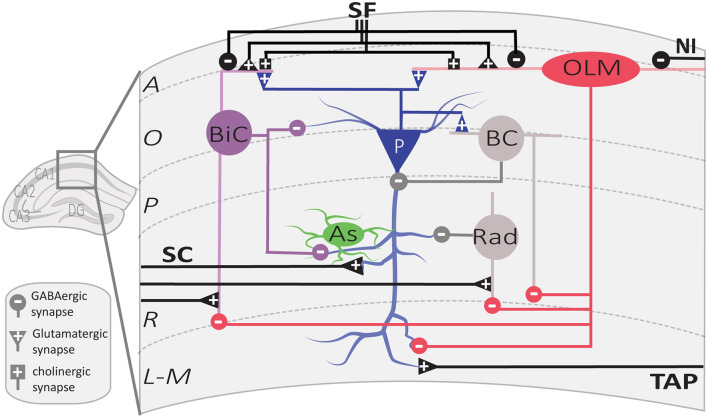
Local circuit of CA1 SOM interneurons. Simplified diagram illustrating the local synaptic circuits of OLM cells and BiCs with other cell types in dorsal CA1 hippocampus. Abbreviations for CA1 layers: A, *alveus*; O, *oriens*; P, *pyramidale*; R, *radiatum*; L-M, *lacunosum-moleculare.* Abbreviations for CA1 afferent inputs: SF, septal fibers; NI, nucleus incertus fibers; SC, Schaffer collateral pathway from CA3 pyramidal cells; TAP, temporo-ammonic pathway from entorhinal cortex. Abbreviations for SOM cell types: (red) OLM, *oriens/lacunosum-moleculare* cell; (violet) BiC, bistratified cell. Abbreviations for other cell types: (blue) P, pyramidal cell; (gray) BC, basket cell; (green) As, astrocyte; (gray) Rad, unidentified *stratum radiatum* interneurons targeted by OLM cells.

The main targets of OLM cell axons are the distal dendrites and spines of PCs (Maccaferri et al., 2000). They also target dendrites of other interneurons, such as bistratified cells, basket cells, and interneurons located in *stratum radiatum* (Schaffer collateral associated cells, perforant path associated cells, and neurogliaform cells; Katona et al., 1999; Elfant et al., 2008; Figure 2). Consequently, CA1 OLM cells provide differential control of PC excitatory afferents: (1) OLM cell activation directly inhibits the distal excitatory inputs of the temporoammonic pathway (TAP); (2) they also inhibit other interneurons, which themselves inhibit the proximal dendrites of CA1 PCs in *stratum* *radiatum*, providing indirect disinhibition of the excitatory inputs from the Schaffer collateral pathway (SC). Hence, OLM cells differentially modulate the excitatory synaptic inputs coming from the entorhinal cortex and CA3 onto PCs (Leao et al., 2012; Katona et al., 2014).

Interestingly, OLM cells express a specific set of proteins regulating their excitatory synaptic inputs. They express a high level of the metabotropic glutamate receptor type 1α (mGluR1α; Ferraguti et al., 2004) which localize perisynaptically at their dendritic synapses (Luján et al., 1996). They also express the postsynaptic adhesion molecule, extracellular leucine-rich repeat fibronectin containing 1 (Elfn1) protein, that interacts with the presynaptic receptor mGluR7, expressed specifically at afferent excitatory synapses of OLM cell (Shigemoto et al., 1996; Sylwestrak and Ghosh, 2012). The Elfn1-mGluR7 interaction facilitates excitatory transmission at these synapses and maximizes excitation in the theta frequency range (4–10 Hz; Sylwestrak and Ghosh, 2012).

The hippocampus displays characteristic oscillatory activities, theta and, gamma oscillations as well as sharp-wave ripples, during exploration and rapid eye movement (REM) sleep (Vanderwolf, 1969; O’Keefe and Nadel, 1978; Buzsaki et al., 1992; Buzsáki, 2002). Thus, the relation of interneuron firing to hippocampal oscillations provides useful functional information (Somogyi et al., 2014). OLM interneurons fire during theta activity at the trough of theta oscillations (Klausberger et al., 2003; Varga et al., 2012; Katona et al., 2014; Somogyi et al., 2014). However, their firing is not coupled to gamma oscillations (Tukker et al., 2007). Also, OLM cell firing is decreased during slow-wave sleep and mostly inhibited during sharp-wave ripples (Klausberger et al., 2003; Varga et al., 2012; Katona et al., 2014). The precise timing of synchronized spikes is crucial for the integration of information because excitatory synapses from CA1 PCs onto SOM interneurons display facilitation on repetitive activation (Ali and Thomson, 1998).

The membrane potential of OLM cells shows intrinsic theta frequency resonance with frequency preferences in theta and low theta ranges (1–5 Hz; Pike et al., 2000). OLM cells display a substantial spike frequency adaptation during sustained discharges (Lacaille and Williams, 1990; Tricoire et al., 2011). These properties constrain OLM cells to delayed low frequency sustained activity (Pouille and Scanziani, 2004). Thus, the facilitation of synaptic activation of SOM interneurons results in gradual recruitment of sustained inhibition of CA1 PCs (Pouille and Scanziani, 2004).

### Bistratified Cells (BiC)

BiCs express the peptide SOM as well as the other interneuron-specific marker, parvalbumin (PV; Pelkey et al., 2017). Their somas are located in or around the CA1 pyramidal cell layer (BiCs; Figure 1). Their vertical aspinous dendrites arborize in *stratum oriens* and *radiatum* (Figure 2; Maccaferri et al., 2000). They receive both feedforward excitation from SC of CA3 PCs and feedback excitation from CA1 PCs (Klausberger et al., 2004). These two major inputs show different short-term dynamics. Repeated stimulation of SC inputs to BiCs show marked and sustained facilitation, whereas similar stimulation of CA1 PCs inputs to BiCs display only an initial transient facilitation (Wierenga and Wadman, 2003; Pouille and Scanziani, 2004). BiCs receive inhibitory synaptic inputs from OLM cells (Leao et al., 2012) and from septal GABAergic neurons (Unal et al., 2018). The designation of BiC comes from the two-layered distribution of their axonal arborizations in the *stratum oriens* and *stratum radiatum* (Figure 2). Accordingly, BiCs strongly inhibit the basal and proximal apical dendrites of CA1 PCs, as well as other interneurons (Halasy et al., 1996; Maccaferri et al., 2000).

The action potentials and subsequent after-hyperpolarization of BiCs are fast, enabling these cells to withstand high frequency stimulation with high temporal reliability (Buhl et al., 1996; Pouille and Scanziani, 2004). In relation to hippocampal oscillatory activity, BiCs fire during theta activity in the trough of theta oscillation (Klausberger et al., 2004). In contrast to OLM cells, BiCs show strong modulation of activity during gamma oscillations and fire during the ascending phase of oscillations (Tukker et al., 2007) In addition, BiCs demonstrate high activity during sharp-wave ripples (Katona et al., 2014). BiCs fast-spiking features and the dynamics of their excitatory and inhibitory inputs influence the way they provide recurrent inhibition of PCs. At the beginning of sustained activation of CA1 PC inputs, BiCs respond with reliable time-locked excitation that shows transient facilitation, resulting in onset-transient inhibition of CA1 PCs proximal dendrites (Wierenga and Wadman, 2003; Pouille and Scanziani, 2004). In contrast, OLM cell firing shows a gradual recruitment during sustained activation of CA1 PC inputs, thereby providing a late-persistent type of inhibition (Pouille and Scanziani, 2004). Thus, BiCs and OLM cells provide recurrent inhibition with distinct dynamics, targeting proximal vs. distal CA1 PC dendrites respectively (Wierenga and Wadman, 2003; Pouille and Scanziani, 2004; Muller and Remy, 2014).

### Hilar Perforant Path (HIPP) Cells

HIPP cells of the dentate gyrus (DG) have their soma and dendritic arborizations in the hilus (Sik et al., 1997; Yuan et al., 2017). Their axonal projections heavily ramify in the outer two-thirds of the molecular layer (Figure 1; Han et al., 1993; Yuan et al., 2017). Although the majority of HIPP axon processes terminate in the DG, some branches traverse the hippocampal fissure and project to the CA1 *stratum lacunosum-moleculare* (Han et al., 1993; Houser, 2007). HIPP cells are mainly excited by granule cell (GC) axons and provide feedback inhibition to distal dendrites of GCs, thereby showing functional similarities with OLM cells (Hosp et al., 2014). HIPPs form functional synapses with GCs, but also with PV interneurons and other HIPP cells. They also rarely connect to CCK hilar commissural associational path (HICAP) interneurons (Savanthrapadian et al., 2014). HIPP provides weak and slow dendritic inhibition (Yuan et al., 2017). However, their inhibition of PV cells is sufficient to regulate their action potential generation and spike timing precision, and hence, control information flow within the DG circuitry (Savanthrapadian et al., 2014).

### Radiatum (R) Cells

Other subtypes of SOM cells, that are sparse in number, are found in *stratum radiatum* (Figure 1; Oliva et al., 2000; Tricoire et al., 2011). One type of SOM interneuron was identified in a transgenic mice line (GFP-expressing Inhibitory Neurons, GIN mice; Oliva et al., 2000). These CA1 SOM interneurons have a cell body in *stratum radiatum* and dendrites that span *stratum oriens* and *stratum radiatum*. Their axonal projections ramify in *stratum lacunosum-moleculare* and these cells were designated *radiatum-lacunosum moleculare* (RLM cells; Figure 1; Oliva et al., 2000). RLM cells show similar firing properties to OLM cells (Oliva et al., 2000). Hence, RLM cells may provide feedforward inhibition of entorhinal cortex input of PCs (Oliva et al., 2000). Another type of SOM cell was identified with the soma, dendrites and axonal projections restricted to *stratum* *radiatum* (SR cells; Figure 1; Oliva et al., 2000; Tricoire et al., 2011). These SOM cells have similar firing properties to OLM cells and may provide feedforward inhibition of Schaffer collateral input to CA1 pyramidal cells (Oliva et al., 2000; Tricoire et al., 2011).

### Projection Cells

Distinct SOM interneurons, in addition to providing local inhibition, have projections to other hippocampal and subcortical areas, where they target many cell types including PCs and GABA interneurons (Gulyas et al., 2003; Katona et al., 2017; Eyre and Bartos, 2019). In CA1, these SOM projecting cells have their soma and dendrites in *stratum oriens*, as well as local axonal arborizations in *stratum oriens* and *stratum radiatum*. Distinct CA1 SOM projection cells are distinguished by their axonal projection targets (Figure 1). Back-projecting (BP) cells have axonal projections to CA3 and DG (Goldin et al., 2007; Katona et al., 2017). Oriens-retrohippocampal projecting (ORP) cells project to the subiculum (Jinno et al., 2007; Melzer et al., 2012). Double-projecting (DP) cells have axons projecting to the septum, as well as to CA3 and DG (Gulyas et al., 2003; Katona et al., 2017; Eyre and Bartos, 2019), subiculum (Goldin et al., 2007; Jinno et al., 2007; Melzer et al., 2012) or the medial entorhinal cortex and contralateral hippocampus (Goldin et al., 2007; Jinno et al., 2007; Melzer et al., 2012; Eyre and Bartos, 2019). Much less is known about the physiological properties of these cell types, although *in vivo* recordings indicate that most projection cells discharge during theta activity in the descending slope or at the trough of theta cycles during movement (Jinno et al., 2007; Katona et al., 2017). Some projection cells fire preferentially on the ascending phase of gamma oscillations (Jinno et al., 2007). Most of them also increase their firing during sharp-wave ripples (Jinno et al., 2007; Katona et al., 2017). Thus, SOM projection cells tend to fire similarly to BiC cells during hippocampal oscillations.

In CA3, DP cells were described with soma in *stratum oriens*, *pyramidale*, and *lucidum* (Gulyas et al., 2003; Jinno et al., 2007; Figure 1). CA3 DP cells have dendrites in *stratum oriens* and *radiatum*, as well as local axons in *stratum oriens*, *pyramidale* and *radiatum* (Jinno et al., 2007). CA3 DP cells have axonal long-range projections that target the septum, as well as CA1 and subiculum (Jinno et al., 2007) and ventral hippocampal areas (Gulyas et al., 2003). CA3 DP cell axonal long-range projections make synaptic contacts with PCs and GABA interneurons (Gulyas et al., 2003; Jinno et al., 2007).

In DG, another type of SOM cell is the hilar cell (HIL, Figure 1). HIL cells have a cell body, dendrites, and local axonal projections in the hilus (Yuan et al., 2017). Their activity is driven by excitatory inputs from GCs and DG mossy cells (Larimer and Strowbridge, 2008; Yuan et al., 2017). HIL cells provide perisomatic inhibition of hilar GABAergic cells and mossy cells and send long-range axonal projections to the medial septum (Gulyas et al., 2003; Larimer and Strowbridge, 2008; Yuan et al., 2017) and contralateral DG (Gulyas et al., 2003; Goldin et al., 2007; Jinno et al., 2007; Eyre and Bartos, 2019). By exerting a powerful inhibition onto local GABAergic and mossy cells, as well as septal neurons, HIL cells can coordinate activities in these areas as a function of GC and mossy cell activation (Larimer and Strowbridge, 2008; Yuan et al., 2017).

## SOM Interneurons Long-Term Synaptic Plasticity

In the central nervous system, two widely studied forms of long-term synaptic plasticity are long-term potentiation (LTP), a long-lasting strengthening of synaptic efficacy, and long-term depression (LTD), a long-lasting weakening of synaptic efficacy (Malenka and Bear, 2004). These forms of synaptic plasticity at hippocampal excitatory synapses are linked to memory storage (Morris, 2003). *In vitro*, several protocols of electrical stimulations, or repetitive pairing of pre- and post-synaptic firing, induce long-term changes of synaptic transmission (Malenka and Bear, 2004; Caporale and Dan, 2008). With electrical stimulation, LTP is generally associated with high frequency afferent stimulation, and LTD with low frequency stimulation. With pre- and post-synaptic pairing, a presynaptic spike preceding the postsynaptic spike within a narrow time window induces LTP, whereas the reverse produces LTD (Feldman, 2012). Although it has been known for some time that blocking GABAergic transmission facilitates the induction of LTP at excitatory synapses (Wigstrom and Gustafsson, 1983) and that afferents of inhibitory interneurons display long-term potentiation (Buzsaki and Eidelberg, 1982), synaptic plasticity at excitatory synapses onto interneurons has recently attracted more attention (Kullmann and Lamsa, 2007; Pelletier and Lacaille, 2008). It is becoming increasingly apparent that hippocampal inhibitory neurons, including SOM interneurons, have highly dynamic activity and express long-lasting changes at their excitatory input synapses and inhibitory output synapses (Maccaferri and McBain, 1996; Perez et al., 2001; Lamsa et al., 2005; Chevaleyre et al., 2006; Kullmann and Lamsa, 2007, 2011; Pelletier and Lacaille, 2008; Vasuta et al., 2015; Udakis et al., 2020). In this section, we will focus on long-term plasticity at excitatory synapses onto hippocampal SOM interneurons (Perez et al., 2001) and later examine its role in hippocampus-dependent memory.

### Excitatory Synapses Onto CA1 SOM Interneurons

Plasticity of excitatory synapses onto SOM interneurons has been characterized the most in CA1 OLM cells (Lacaille et al., 1987; Maccaferri and McBain, 1996; Perez et al., 2001; Lamsa et al., 2007). Excitatory synapses made by CA1 PC axons that feedback on CA1 SOM interneurons (notably OLM cells) are composed of Ca^2+^ permeable AMPA receptors (CP-AMPARs). These synapses show inward rectification of their current-voltage (I–V) relationship, display short-term facilitation with repeated stimulation, and are inhibited by mGluR2/3 (Croce et al., 2010). In contrast, excitatory synapses made by axons of CA3 PCs that feed-forward onto CA1 SOM interneurons (OLM cells) are composed of Ca^2+^ impermeable AMPARs (CI-AMPARs), and these synapses show linear I–V relationships, display short-term depression with repeated stimulation, and are mGluR2/3-insensitive (Croce et al., 2010). These input-specific properties of excitatory synapses differentially control the SOM interneuron output firing, resulting in gradual sustained recruitment of evoked firing with repetitive feedback input activation, and transient evoked firing with repetitive feedforward input activation of SOM cells (Croce et al., 2010). It is interesting to note that input-specific rules of excitatory synapses also occur for other types of interneurons in CA3 *stratum lucidum* (SL) but with the opposite organization (Toth and McBain, 1998). Thus, excitatory synapses onto single SOM cells originate from multiple sources and display afferent-specific mechanisms. In addition, these afferent-specific mechanisms differ from those in other types of interneurons.

### Long-Term Potentiation at Excitatory Synapses Onto SOM Interneurons

The synapse between CA1 PC axons and SOM interneurons, notably BiC and OLM cells, express a Hebbian form of LTP that requires the coincident activity of both pre- and postsynaptic neurons for induction (Perez et al., 2001; Lapointe et al., 2004; Vasuta et al., 2015). Multiple lines of evidence indicate that this SOM interneuron LTP is not due to passive propagation of di-synaptic LTP at Schaffer collateral synapses on CA1 pyramidal cells (SC-CA1 synapses; McBain et al., 1999). First, SOM interneuron LTP is insensitive to the NMDA receptor blocker AP-5 (Perez et al., 2001), unlike LTP at SC-CA1 PC synapses (Morris et al., 1986). Second, LTP is induced directly at putative single fiber synapses onto SOM interneurons by using minimal stimulation (Perez et al., 2001; Lapointe et al., 2004; Vasuta et al., 2015). Finally, interfering with Ca^2+^ influx in the postsynaptic SOM interneuron prevents LTP induction (Lapointe et al., 2004).

**Figure 3:**
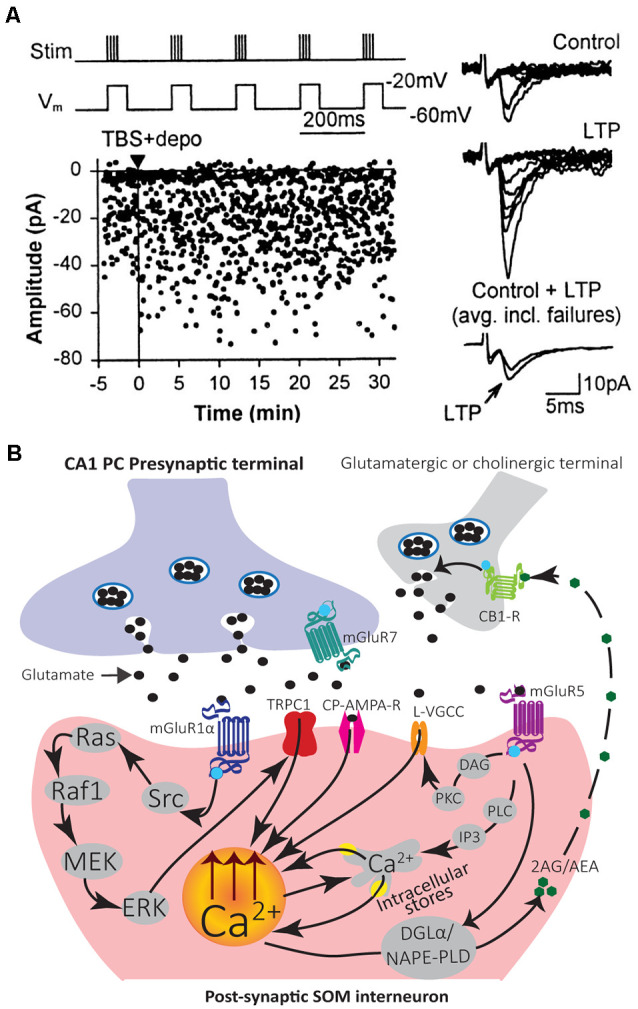
Mechanisms of induction and modulation of Hebbian LTP in SOM interneurons. **(A)** Graph of EPSC amplitude from a representative cell showing LTP after TBS paired with depolarization (TBS + depo; protocol shown above and delivered at time indicated by black triangle and vertical line). Examples of control and potentiated EPSCs (10 consecutive responses) at 30 min after induction (*Right*). Superimposed average EPSCs (bottom traces; *n* = 64 responses each, failures included) from control and 30 min after induction also show LTP. Adapted from Perez et al. (2001). **(B)** Diagram of LTP induction mechanisms leading to postsynaptic Ca^2+^ elevation in SOM interneurons. Stimulation of the presynaptic terminal releases glutamate that activates postsynaptic CP-AMPAR and mGluR1α. Activation of mGluR1α stimulates the Src signaling cascade leading to ERK activation. ERK leads to postsynaptic Ca^2+^ rise *via* activation of non-selective cationic channels TRPC1 and Ca^2+^ entry, as well as mobilization of Ca^2+^ from intracellular stores. A retrograde signaling mechanism may involve eCBs. Synaptic activation of mGluR1/5 stimulates synthesizing enzymes DGLα and/or NAPE-PLD to produce the eCBs 2-AG and/or AEA. 2-AG/AEA act retrogradely to activate CB1Rs on glutamatergic or cholinergic terminals and potentiate transmission. A potential mechanism of LTP modulation may involve activation of mGluR5 that produces postsynaptic Ca^2+^ rises *via* two pathways. First, mGluR5 activates the PLC/IP3 pathway which leads to Ca^2+^ release from internal stores. Second, mGluR5 activates DAG-PKC pathway to potentiate L-type VGCC function and may regulate LTP induction.

This LTP is considered Hebbian since it is induced by presynaptic theta-burst stimulation (TBS) paired with postsynaptic depolarization (Figure 3), but not by presynaptic stimulation alone nor postsynaptic depolarization alone (Perez et al., 2001). Hebbian LTP is expressed as a decrease in failure rates of EPSCs and an increase in the potency of EPSCs (amplitude of EPSCs excluding failures; Perez et al., 2001; Lapointe et al., 2004). It is also accompanied by a change in paired-pulse facilitation and the coefficient of variation of EPSCs, parameters associated with presynaptic changes (Lapointe et al., 2004). Thus, Hebbian LTP may be expressed by both pre- and post-synaptic mechanisms.

Hebbian LTP in SOM interneurons shows afferent input specificity. Pairing presynaptic TBS with postsynaptic depolarization elicits LTP at synapses between CA1 PCs and SOM interneurons, but not at the synapses between CA3 PCs and SOM interneurons (Croce et al., 2010). Also, it displays cell-type specificity. Pairing presynaptic TBS with postsynaptic depolarization evoked LTP in SOM interneurons (BiC and OLM cells; Perez et al., 2001; Lapointe et al., 2004; Vasuta et al., 2015) but not in PV interneurons (Vasuta et al., 2015) nor in unidentified interneurons in *stratum radiatum* (Perez et al., 2001).

Induction of Hebbian LTP in SOM interneurons involves a very different signaling cascade than LTP in PCs (Figure 3). Most notably, Hebbian LTP in SOM interneurons does not involve NMDA receptors (Perez et al., 2001), but requires the activation of mGluR1α receptors, a glutamate metabotropic receptor highly expressed in SOM interneurons (Perez et al., 2001; Kougioumoutzakis et al., 2020). Pharmacologically blocking mGluR1α, or genetically deleting mGluR1 (mGluR1^−^/^−^ mice), prevents Hebbian LTP induction, indicating a crucial role of mGluR1α (Perez et al., 2001; Lapointe et al., 2004; Topolnik et al., 2005; Vasuta et al., 2015). During induction of Hebbian LTP, the activation of postsynaptic mGluR1α stimulates Src and extracellular signal-regulated kinase (ERK) pathways, causing the opening of transient receptor potential (TRP) channels and Ca^2+^ influx, as well as Ca^2+^ release from intracellular stores (Topolnik et al., 2006; Figure 3). TRPC1 interaction with mGluR1α in SOM interneuron dendrites mediates a mGluR1α-dependent slow EPSC in SOM interneurons, supporting the importance of TRP channels in SOM interneurons LTP (Kougioumoutzakis et al., 2020).

Endocannabinoid (eCB) signaling mainly mediates short- and long-term depression of excitatory and inhibitory transmission (Chevaleyre et al., 2006). In addition, synaptic activation of group I mGluRs (mGluR1/5) is a major pathway for the production of eCBs (Chevaleyre et al., 2006). Interestingly in SOM interneurons, eCBs may be involved as a retrograde messenger in LTP (Friend et al., 2019). SOM interneurons express the endocannabinoid-synthesizing enzyme diacylglycerol lipase α (DGLα) and n-acylphosphatidylethanolamine phospholipase D (NAPE-PLD; Friend et al., 2019). Moreover, inhibition of cannabinoid type 1 receptor (CB1R) prevents LTP in SOM interneurons (Friend et al., 2019). Thus, synaptic activation of mGluR1/5 may lead to the production of eCBs (anandamide, AEA; 2-arachadonyl glycerol, 2-AG) that act retrogradely on presynaptic CB1Rs to potentiate synaptic transmission (Figure 3; Friend et al., 2019).

Activation of mGluR5 in SOM interneurons (OLM cells) also elicits postsynaptic Ca^2+^ rises from intracellular stores release, independent of Src-ERK activation (Topolnik et al., 2006). Moreover, this type of Ca^2+^ signaling is not involved directly in Hebbian LTP induction (Topolnik et al., 2006). Pharmacological activation of mGluR5 is sufficient to induce LTP at excitatory synapses onto SOM interneurons (OLM cells), indicating multiple types of LTP linked to mGluRs in SOM interneurons (Le Vasseur et al., 2008). In addition, local mGluR5 activation by agonist application or high frequency synaptic stimulation leads to sustained enhancement of action potential evoked Ca^2+^ transients in dendrites of SOM interneurons (OLM cells; Topolnik et al., 2009). This augmentation of postsynaptic Ca^2+^ transients is expressed as a selective potentiation of L-type voltage-gated calcium channels (VGCCs) function and controlled by mGluR5-mediated intracellular Ca^2+^ release as well as protein kinase C (PKC) activation (Figure 3; Topolnik et al., 2009). This activity-dependent regulation of VGCCs by mGluR5 may serve as a mechanism for positively regulating Hebbian synaptic plasticity of SOM interneurons (Topolnik et al., 2009).

In pyramidal cells, GABA_B_Rs mediate a slow K ^+^ -mediated inhibitory postsynaptic current (sIPSC; Dutar and Nicoll, 1988; Degro et al., 2015) and promote excitatory synapses LTP *via* a presynaptic disinhibition mechanism (Davies et al., 1991; Mott and Lewis, 1991). In SOM interneurons, GABA_B_Rs are also highly expressed in dendrites. But instead of activating postsynaptic K^+^ currents, GABA_B_Rs inhibit postsynaptic L-type VGCCs in SOM interneurons (Booker et al., 2018). By negatively regulating VGCCs, GABA_B_Rs inhibit Hebbian LTP in SOM interneurons (Booker et al., 2018). Thus, GABA_B_R-mediated inhibition of L-type VGCCs provides a mechanism for negative regulation of Hebbian synaptic plasticity of SOM interneurons. Interestingly, excitatory and inhibitory synaptic inputs onto SOM interneurons are inhibited presynaptically by GABA_B_Rs, as well as SOM interneuron inhibitory synapses onto PCs (Booker et al., 2020). Thus, GABA_B_R activation may also functionally uncouple SOM interneurons from the CA1 network (Booker et al., 2020).

### Persistent Long-Term Potentiation at Excitatory Synapses Onto SOM Interneurons

LTP in principal cells is divided into two phases: an early phase (early LTP, E-LTP) that is induced by brief high frequency stimulation, lasts several minutes to hours and depends on post-translational mechanisms; and a late phase (late LTP, L-LTP) that requires repetitive high frequency stimulation, lasts several hours to days and depends on new gene expression and protein synthesis (Kandel, 2001; Abraham et al., 2019). Although the study of late LTP has mainly focused on excitatory synapses onto principal cells (Malenka and Bear, 2004), recent studies revealed that excitatory synapses onto CA1 SOM interneurons can also undergo L-LTP (Ran et al., 2009, 2012; Artinian et al., 2019).

**Figure 4:**
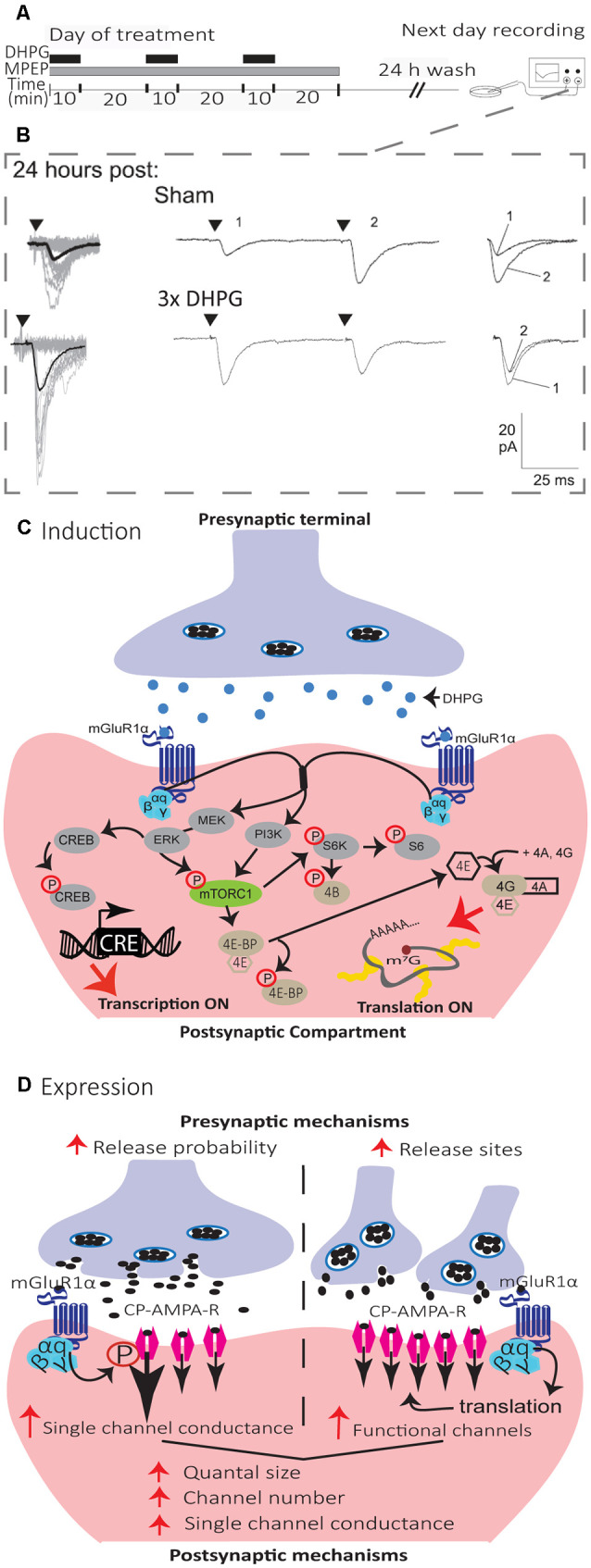
Mechanisms of induction and expression of L-LTP_mGluR1_ in SOM interneurons. **(A)** Schematics of induction and recording protocol. Cultured hippocampal slices were treated with repetitive (3×) applications of the mGluR1/5 agonist (DHPG, 5 μM, black bars) in the presence of the mGluR5 antagonist (MPEP, 25 μM, gray bar). On the next day, after a 24 h wash-out, whole cell recordings were obtained from visually identified CA1 SOM interneurons in agonist- or sham-treated slices. **(B)** Representative EPSCs evoked by minimal stimulation at 24 h after sham-treatment (top), repetitive (3×; bottom) mGluR1 agonist-stimulation, showing larger responses after repetitive treatment. Left, superimposed 20 successive events (EPSCs + failures; gray) with average EPSC (including failures; solid black line) of 100 events. Middle, average of EPSC pairs (100 events) evoked by paired-pulse stimulation (50 ms interstimulus interval), showing loss of paired-pulse facilitation after repetitive treatment. Right, superimposed first and second EPSCs of average pair. Black triangles indicate the time of stimulation. Adapted from Ran et al. (2009). **(C)** Diagram of L-LTP_mGluR1_ induction mechanisms. Repeated stimulation of mGluR1 by DHPG engages PI3K and MEK/ERK signaling pathways to phosphorylate mTORC1. Activation of mTORC1 leads to initiation of eIF4E-mediated mRNA translation *via* to pathways: (1) ribosomal S6 protein kinase (S6K) stimulation of S6 phosphorylation; and (2) phosphorylation of 4E-BPs repressors proteins which release eIF4E. Subsequently, eIF4E associates with eIF4A and eIF4G to form the cap-binding complex, eIF4F, which initiates translation. Activation of MEK–ERK signaling by repeated mGluR1 stimulation also leads to phosphorylation of CREB to control CRE-dependent gene expression. **(D)** Diagram of L-LTP_mGluR1_ expression mechanisms. The maintenance of L-LTP_mGluR1_ involves both pre- and post-synaptic modifications. At the presynaptic level, transmitter release is increased (increase in release probability and/or addition of functional release site). At the postsynaptic level, postsynaptic responsiveness is increased (recruitment of functional receptors and increase in single-channel conductance).

In hippocampal slice culture, repetitive stimulation of mGluR1, by the repeated application of the mGluR1/5 agonist (RS)-3, 5-dihydroxyphenylglycine (DHPG), in the presence of the mGluR5 antagonist 2-methyl-6-(phenylethynyl) pyridine (MPEP), induces a persistent potentiation of excitatory synapses onto SOM interneurons that can last at least 24 h, termed mGluR1-dependent late LTP (L-LTP_mGluR1_; Figure 4; Ran et al., 2009, 2012; Artinian et al., 2019). L-LTP_mGluR1_ is prevented by inhibitors of transcription and translation, indicating that it is dependent on new gene expression and protein synthesis (Figure 4; Ran et al., 2009). L-LTP_mGluR1_ is also induced in acute hippocampal slices by repeated mGluR1 stimulation with DHPG, or by repetitive theta-burst stimulation of afferents (Artinian et al., 2019). Interestingly, using *ex vivo* whole-cell recordings in acute slices 24 h after contextual fear conditioning, training was found to induce mGluR1-mediated, translation-dependent L-LTP at SOM interneuron excitatory synapses, indicating that L-LTP_mGluR1_ may be linked to hippocampus-dependent memory (Artinian et al., 2019).

Mechanistically, L-LTP_mGluR1_ shares many features of Hebbian LTP. Repetitive stimulation of mGluR1 by DHPG during action potentials blockade with tetrodotoxin prevent L-LTP_mGluR1_, suggesting a Hebbian induction (Ran et al., 2012). L-LTP_mGluR1_ is also NMDAR independent, and prior induction of L-LTP_mGluR1_ occludes induction of Hebbian LTP, indicating that they share similar mechanisms (Ran et al., 2012).

The mechanisms of induction of L-LTP_mGluR1_ (Figure 4) requires activation of mGluR1α, as it is prevented by the selective antagonist LY367385 (Ran et al., 2009; Artinian et al., 2019). Activation of mGluR1α stimulates both phosphoinositide 3-kinase (PI3K) and mitogen-activated protein kinase kinase (MEK)/extracellular signal-regulated kinase (ERK) pathways resulting in mTORC1 phosphorylation. In turn, mTORC1 activates ribosomal protein S6 kinase (S6K) and S6 phosphorylation, as well as phosphorylation of eIF4E-binding protein (4E-BP), to stimulate eIF4E-dependent translation necessary for L-LTP_mGluR1_ (Ran et al., 2009; Artinian et al., 2019). In addition activation of mGluR1α stimulates phosphorylation of cAMP response element-binding protein (CREB) *via* ERK signaling, to activate CREB-dependent transcription also necessary for L-LTP_mGluR1_ (Ran et al., 2012). A cell-specific conditional knock-out in SOM interneurons of the Regulatory-Associated Protein of mTOR (Raptor) gene, a necessary component of mTORC1, reduces mTORC1 activity and prevents L-LTP_mGluR1_ (Artinian et al., 2019). Conversely, cell-specific conditional heterozygous knock-out in SOM interneurons of the Tuberous Sclerosis Complex 1 (TSC1) gene, a repressor of mTORC1, increases mTORC1 activity in SOM interneurons and facilitates L-LTP_mGluR1_ by lowering the threshold for induction (Artinian et al., 2019). Knock-out of the 4E-BP gene, which removes the repression of eIF4E-dependent translation, similarly causes a facilitation of L-LTP_mGluR1_ by lowering the threshold for induction (Ran et al., 2009), further highlighting the critical role of mTORC1-dependent translation in L-LTP_mGluR1_ (Figure 4).

The mechanisms of expression of synaptic plasticity in central neurons are varied and complex (Citri and Malenka, 2008). In SOM interneurons, L-LTP_mGluR1_ expression may involve both pre- and postsynaptic mechanisms, not occurring necessarily jointly (Figure 4; Ran et al., 2009, 2012; Artinian et al., 2019). In slice cultures, L-LTP_mGluR1_ is expressed by an increase in potency of EPSCs evoked by minimal stimulation (Ran et al., 2009; Artinian et al., 2019), and in amplitude of miniature EPSC (mEPSCs; Ran et al., 2009), suggesting postsynaptic mechanisms of expression. L-LTP_mGluR1_ in slice cultures is accompanied by a reduction in the paired-pulse ratio of EPSCs evoked by minimal stimulation (Ran et al., 2009; Artinian et al., 2019) and by an increase in mEPSC frequency (Ran et al., 2009), suggesting presynaptic mechanisms of expression. Moreover, quantal analysis of EPSCs evoked by minimal stimulation in slice culture indicated an increase in quantal content and quantal size during L-LTP_mGluR1_, consistent with coordinated pre- and post-synaptic changes (Ran et al., 2012). The increase in quantal content may result from increased release probability or new release sites (Ran et al., 2012). Conforming with the increase in quantal size, peak-scaled nonstationary fluctuation analysis of mEPSCs indicated that an increase in both single-channel conductance and number of functional receptors contribute to the increase in the postsynaptic response during L-LTP_mGluR1_ (Ran et al., 2012). In acute slices, L-LTP_mGluR1_ induced by DHPG is expressed by an increase in EPSC potency, but no change in paired-pulse ratio, whereas L-LTP_mGluR1_ induced by TBS stimulation is expressed by an increase in EPSC potency and a decrease in paired-pulse ratio (Artinian et al., 2019). Similarly, L-LTP_mGluR1_ induced by contextual fear conditioning is expressed by an increase in spontaneous EPSC amplitude and frequency, as well as an increase in potency of EPSCs evoked by minimal stimulation but no change in the paired-pulse ratio (Artinian et al., 2019), indicating that pre- and post-synaptic expression mechanisms do not always occur jointly during L-LTP_mGluR1_.

### Other Types of Synaptic Plasticity in SOM Interneurons

Interestingly, another form of plasticity called anti-Hebbian LTP is induced at excitatory synapses onto CA1 OLM interneurons by pairing presynaptic stimulation with postsynaptic membrane hyperpolarization (Kullmann and Lamsa, 2007). The anti-Hebbian LTP is NMDAR-independent and dependent on CP-AMPARs. This form of LTP is not specific to SOM interneurons and can be induced in fast-spiking PV interneurons (axo-axonic cells and basket cells; Kullmann and Lamsa, 2007). The diversity in types of long-term plasticity at SOM interneuron synapses in the hippocampal CA1 region suggests multiple roles in long-lasting regulation of the CA1 network.

In CA3, excitatory synapses onto interneurons display different types of synaptic plasticity including NMDA-dependent LTP and NMDAR-independent LTD (Laezza et al., 1999; Laezza and Dingledine, 2004). However, whether synaptic plasticity occurs at synapses onto CA3 SOM interneurons remains to be determined.

In DG, however, excitatory synapses onto both types of SOM interneurons, HIPP and HIL cells, show long-term synaptic plasticity (Yuan et al., 2017). In these cases, plasticity is induced by the application of an associative burst frequency stimulation at 30 Hz (aBFS) of afferents paired with postsynaptic action potentials, a stimulation protocol aimed at mimicking fast rhythmic neuronal network activity patterns at gamma (30–100 Hz) frequencies in DG. Remarkably, aBFS induces long-lasting depression (LTD) of excitatory synapses from GC onto HIPP cells, but LTP of excitatory synapses from GC and mossy cells onto HIL cells (Yuan et al., 2017). The increase and the decrease in the failure rate of synaptic transmission accompanying LTD in HIPP cells and LTP in HIL cells, respectively, suggest that both phenomena involve presynaptic expression mechanisms (Yuan et al., 2017). These results indicate that long-term synaptic plasticity in DG HIPP cells differ from that in CA1 OLM cells. Thus, although both cell types provide local dendritic feedback inhibition, their synapses from excitatory inputs display different types of long-term changes, indicating region-specific plasticity properties.

## Regulation of CA1 Network Metaplasticity by Plasticity of Excitatory Synapses of SOM Interneurons

As mentioned previously, early work established that afferents of inhibitory interneurons express long-term potentiation (Buzsaki and Eidelberg, 1982). Coupled with the finding that pharmacological inhibition of GABAergic transmission facilitated the induction of LTP at PC excitatory synapses (Wigstrom and Gustafsson, 1983), this led to the concept that plasticity at excitatory synapses in interneurons increase inhibition of PCs and dampen LTP at PC excitatory synapses. However, more recent work indicates that multiple types of synaptic plasticity at excitatory synapses onto inhibitory interneurons have more complex actions in hippocampal networks (Kullmann and Lamsa, 2007; Pelletier and Lacaille, 2008).

### Long-Term Plasticity of Excitatory Synapses Controls SOM Interneuron Firing and Synaptic Output

Dendrite-projecting SOM interneurons provide efficient suppression of CA1 PC dendritic excitatory synaptic inputs and synaptically-evoked burst firing (Lovett-Barron et al., 2012). *In vivo* during spatial navigation, dendritic inhibition by SOM interneurons suppresses the firing of PCs in their place fields, as well as PC burst firing (Royer et al., 2012). But dendritic inhibition is not static, and the recruitment of recurrent dendritic inhibition in PCs is dynamic during short trains of stimulation (Pouille and Scanziani, 2004). At the onset of a series of stimuli, soma- and proximal dendrite-targeting recurrent inhibition are elicited. Later in the series of stimuli, recurrent inhibition is evoked in the distant dendritic regions. Thus, during repetitive activation of PCs, recurrent inhibition switches from transient somatic inhibition to late persistent dendritic inhibition (Pouille and Scanziani, 2004). These dynamic changes in dendritic inhibition are due in part to a late and persistent recruitment on SOM interneuron (OLM cell) firing during short trains of CA1 PC stimulation.

Induction of Hebbian LTP during voltage clamp recordings requires the pairing of presynaptic stimulation with postsynaptic depolarization (Perez et al., 2001). However, during current clamp recordings, presynaptic TBS stimulation alone is sufficient to activate mGluR1a and evoke EPSPs that trigger postsynaptic firing of action potentials in SOM interneurons, and thus, induce Hebbian LTP (Vasuta et al., 2015). Using the TBS induction protocol in slices permits the assessment of the functional impact of Hebbian LTP in SOM interneurons. As synaptic efficacy is improved during Hebbian LTP at excitatory synapses in SOM interneurons, the synaptically-evoked firing of SOM interneurons should show long-lasting increases after Hebbian LTP. Indeed, short trains of afferents stimulation in slices elicit two patterns of evoked firing in SOM interneurons (BiC and OLM cells): an onset-transient firing consistent with activation of CA3 afferents; or a late-persistent firing consistent with activation of CA1 afferents (Croce et al., 2010). After application of the TBS induction protocol for Hebbian LTP, onset-transient responses are unchanged but late-persistent firing responses of SOM interneurons show long-term increases (Croce et al., 2010). These long-term changes in synaptically-evoked firing are prevented by the application of the selective mGluR1α antagonist LY367385 during the TBS induction (Croce et al., 2010). These results suggest that Hebbian LTP at excitatory synapses translates into an increase in output firing of SOM interneurons (Croce et al., 2010).

**Figure 5:**
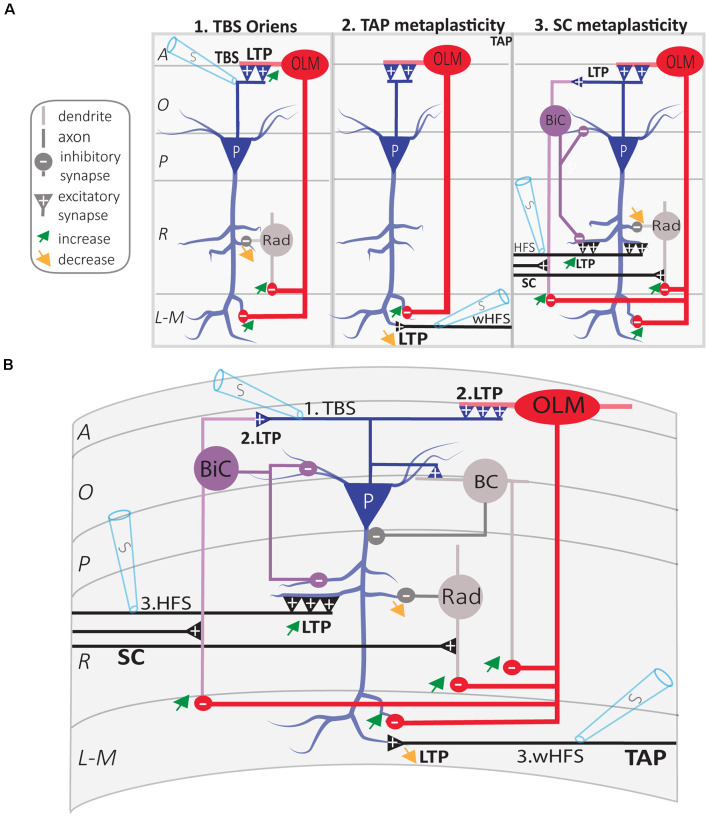
Regulation of CA1 network metaplasticity by plasticity at excitatory synapses of SOM interneurons. **(A)** Diagrams of synaptic plasticity of SOM interneurons and regulation of CA1 network. (A1) Theta burst stimulation (TBS) at the O/A border induces LTP of CA1 PC synapses onto SOM interneurons (OLM cell). LTP at input synapses results in an increased firing of OLM cell, which leads to increased postsynaptic inhibition of (i) PC distal dendrites and (ii) *radiatum* interneurons (rad). This leads to the increased inhibition of distal dendrites in *stratum lacunosum-moleculare* (L-M), and disinhibition of more proximal dendrites in *stratum radiatum* (R). (A2) LTP of excitatory inputs of OLM cell results in TA pathway metaplasticity. Because of distal dendritic inhibition increase, tetanization of the temporoammonic (TA) pathway produces less LTP at TA-PC synapses. (A3) LTP of excitatory inputs of OLM cell results in SC pathway metaplasticity. Because of proximal dendritic disinhibition, tetanization of Schaffer collaterals (SC) produces larger LTP at SC-PC synapses. As electrically induced TBS also produces LTP at PC-BiC synapses, the overall results on BiC inhibition of PCs remain undetermined. **(B)** Integration of synaptic mechanisms described above: 1. TBS of PC axons; 2. LTP at PC-OLM synapses results in more inhibition of distal PC dendrites and disinhibition of proximal PC dendrites; the end-result of LTP at PC-BiC synapses on BiC inhibition of PC remains undetermined. 3. Metaplastic changes of CA1 circuit: increased LTP at SC-PC synapses and decreased LTP at TA-PC synapses. Abbreviations for CA1 layers: A, *alveus*; O, *oriens*; P, *pyramidale*; R, *radiatum*; L-M, *lacunosum-moleculare.* Abbreviations for CA1 afferent inputs: SC, Schaffer collateral pathway; TAP, temporo-ammonic pathway. Abbreviations for SOM cell types: (red) OLM, o*riens/lacunosum-moleculare* cell; (violet) BiC, bistratified cell. Abbreviations for other cell types: (blue) P, pyramidal cell; (gray) BC, basket cell; (gray) Rad, unidentified *stratum radiatum* interneurons. Other Abbreviation: S, stimulation electrode.

Using a similar approach during the whole-cell recording of inhibitory postsynaptic currents (IPSCs) in CA1 pyramidal cells, the TBS induction protocol for Hebbian LTP produces long-term increases in postsynaptic inhibitory responses in pyramidal cells (Lapointe et al., 2004). The long-term increase in inhibition is not accompanied by any change in excitatory postsynaptic response, and is prevented in mGluR1 knockout mice that lack Hebbian LTP in SOM interneurons (Lapointe et al., 2004). These findings suggest that Hebbian LTP at excitatory synapses of SOM interneurons translates in long-term increases in SOM cell firing and pyramidal cell inhibition (Figure 5).

### Long-Term Plasticity of SOM Interneuron Excitatory Synapses Controls CA1 Network Metaplasticity

Synaptic plasticity is bidirectionally modulated by prior cellular and/or synaptic activity, a phenomenon called metaplasticity (plasticity of synaptic plasticity; Bear, 1995). In the hippocampus, long-term plasticity at SC-CA1 PC synapses is regulated by multiple metaplastic mechanisms, including both homo- and hetero-synaptic processes (Abraham, 2008). In CA1, the dendrite-projecting SOM interneurons have emerged as central players in the regulation of the local network metaplasticity. Optogenetic activation of CA1 SOM interneurons (OLM cells) dampens information flow from the entorhinal cortex through the TA pathway *via* direct inhibition of CA1 PCs distal dendrites, and facilitates information flow from CA3 through the SC pathway *via* inhibition of other inhibitory interneurons (Leao et al., 2012). In addition, CA1 OLM cells regulate the metaplasticity of TA and SC pathways (Figure 5). Optogenetic activation of OLM cells during LTP induction decreases LTP at TA-PC synapses, whereas it facilitates LTP at SC-PC synapses (Leao et al., 2012).

Since Hebbian LTP at excitatory synapses onto SOM interneurons results in long-term changes in their output firing and inhibition of pyramidal cells, it should also result in long-term regulation of plasticity at SC and TA synapses onto PCs (Figure 5). Indeed, application of the TBS induction protocol for Hebbian LTP in SOM interneurons does not affect basal transmission at SC-PC synapses, but increases the magnitude of LTP at SC-PC synapses elicited 30 min later (Vasuta et al., 2015). This facilitation of LTP at SC-PC synapses is prevented by optogenetic silencing of SOM interneurons during the TBS induction protocol, or by the mGluR1α antagonist LY367385, suggesting that Hebbian LTP in SOM interneurons result in long-term upregulation of plasticity of SC-PC synapses (Vasuta et al., 2015). Likewise, application of the induction protocol for mGluR1-dependent late LTP (L-LTP_mGluR1_) in SOM interneurons facilitates LTP of SC-PC synapses elicited 2 h later (Artinian et al., 2019). This persistent facilitation of LTP at SC-PC synapses is absent in mice with conditional knock-out of *Rptor* in SOM interneurons that lack L-LTP_mGluR1_, suggesting that long-term upregulation of plasticity of SC-PC synapses lasting hours results from late-LTP at excitatory synapses onto SOM interneurons (Artinian et al., 2019). De-phosphorylation of the eukaryotic initiation factor 2 α subunit (eIF2α) by synaptic activity is a key regulator of mRNA translation and late-LTP in PCs (Costa-Mattioli et al., 2007). Knock-in mice with a mutated non-phosphorylatable eIF2α (eIF2a^S51A^) show facilitation of late-LTP in CA1 PCs (Costa-Mattioli et al., 2007). Interestingly, conditional knock-in of eIF2α^S51A^ specifically in SOM interneurons, upregulates mRNA translation in SOM interneurons, reduces inhibitory synaptic transmission in CA1 PCs, and facilitates induction of L-LTP at SC-PC synapses (Sharma et al., 2020). Altogether these findings suggest that synaptic plasticity at excitatory synapses of SOM interneurons acts as a long-term metaplasticity switch at SC-PCs synapses *via* disinhibition (Figure 5).

Similarly, long-term plasticity at excitatory synapses of SOM interneurons may also regulate metaplasticity of TA synapses of PCs. Application of the TBS induction protocol for Hebbian LTP in SOM interneurons does not affect basal transmission at TA-PC synapses but decreases the magnitude of LTP that is elicited 30 min later at these synapses (Sharma et al., 2020). Importantly, this down-regulation of LTP at TA-PC synapses is increased in mice with conditional knock-in of the non-phosphorylatable eIF2a^S51A^ in SOM interneurons that result in upregulated mRNA translation in these cells and impaired inhibition of PCs (Sharma et al., 2020). Thus, long-term plasticity at excitatory synapses of SOM interneurons may act as a bidirectional long-term metaplasticity switch in the CA1 network to differentially regulate long-term plasticity of SC and TA synapses of PCs (Figure 5).

## SOM Interneurons in Hippocampus-Dependent Learning and Memory

At the behavioral level, the dendritic inhibition mediated by SOM interneurons plays a key role in hippocampus-dependent learning and memory (Lovett-Barron et al., 2014). The contribution of SOM interneurons has been studied particularly in the dorsal part of CA1, in relation to the encoding of spatial and contextual memory by PCs.

A combination of *in vivo* calcium imaging with pharmacogenetic and optogenetic manipulations of SOM interneurons in the CA1 hippocampus of mice revealed that dendritic inhibition by SOM interneurons is necessary for contextual fear memory formation (Lovett-Barron et al., 2014). During contextual conditioning, aversive stimuli activate, *via* septal cholinergic inputs, SOM interneurons that target PC dendrites. The activation of SOM interneurons leads to inhibition of PC distal dendrites that receive aversive sensory excitation from the entorhinal cortex (Lovett-Barron et al., 2014). Inactivating dendrite-targeting SOM interneurons during aversive stimuli increases PC responses and prevents fear learning (Lovett-Barron et al., 2014). Thus, activation of dendritic inhibition by SOM interneurons may be a mechanism for the exclusion of aversive stimuli from hippocampal contextual representations that is necessary during fear learning (Lovett-Barron et al., 2014).

Interestingly, GABAergic cells from the brainstem nucleus incertus (NI) selectively inhibit hippocampal SOM interneurons directly, and indirectly by inhibiting septal excitatory inputs to SOM interneurons (Szonyi et al., 2019). NI GABAergic inputs to the hippocampus are activated by relevant salient environmental stimuli *in vivo* (Szonyi et al., 2019). In addition, optogenetic manipulations of NI GABAergic neurons during contextual fear conditioning modify the strength of contextual fear memory: activation of NI GABA neurons impairs, whereas inhibition improves contextual fear memory (Szonyi et al., 2019). Thus, SOM interneuron gating of PC TA sensory inputs during contextual learning may be regulated by the brainstem NI inhibitory system.

### Long-Term Plasticity of SOM Interneuron Excitatory Synapses in Learning and Memory

Cell-specific transgenic mouse approaches were used to test whether there is a functional role of long-term plasticity at SOM interneuron excitatory synapses in hippocampal learning and memory (Artinian et al., 2019). Downregulation of mTORC1 activity was achieved by cell-specific conditional knock-down of the gene for the essential mTORC1 component *Rptor* in SOM interneurons, whereas upregulation of mTORC1 activity was attained by cell-specific conditional knock-down of the mTORC1 repressor *Tsc1* gene*.* At the behavioral level, loss of mTORC1 function specifically in SOM interneurons impaired contextual fear and spatial long-term memories, but spared sensory-motor gating, hippocampus-dependent short-term contextual memory, and hippocampus-independent long-term auditory-cued fear memory (Artinian et al., 2019). In contrast, upregulation of mTORC1 activity specifically in SOM interneurons augmented spatial and contextual fear memories, and impaired discrimination (Artinian et al., 2019).

As mentioned before, at the cellular level, bidirectional regulation of mTORC1 activity in SOM interneurons differentially regulates mGluR1-mediated late-LTP at SOM interneurons excitatory synapses, whereas at the network level, the SOM interneuron late-LTP induction protocol upregulates metaplasticity of the SC pathway in PCs, in a mTORC1-dependent manner. Moreover, using *ex vivo* whole-cell recordings after training, contextual fear learning was found to persistently increase the efficacy of excitatory synapses of SOM interneurons *via* mGluR1 and mTORC1. These findings link mTORC1 to learning-induced long-term plasticity of SOM interneuron excitatory synapses, regulation of CA1 network metaplasticity, and hippocampal long-term memory consolidation (Artinian et al., 2019). Thus, long-term plasticity at SOM interneuron excitatory synapses may play a role in spatial/contextual information encoding by CA1 PCs, by promoting on a long timescale the internal representations by the hippocampal CA3 pathway while dampening external representations *via* the extrahippocampal entorhinal inputs (Artinian et al., 2019).

Recent work manipulating another pathway to enhance memory, de-phosphorylation of the translation initiation factor eIF2α (Costa-Mattioli et al., 2007), further support the idea that SOM interneurons synaptic plasticity is important for memory formation (Sharma et al., 2020). Contextual fear learning reduces the phosphorylation of eIF2α in hippocampal PCs and SOM interneurons, but not in PV interneurons (Sharma et al., 2020). Moreover, cell-specific conditional knock-in of the non-phosphorylatable eIF2α^S51A^ in PCs or in SOM interneurons upregulates general mRNA translation in these cells and is sufficient to increase long-term contextual fear memory (Sharma et al., 2020). Silencing CA1 SOM interneurons, using the inhibitory designer receptor exclusively activated by designer drug (DREADD), during the consolidation of fear memory, reverses the increase in contextual fear memory in the SOM interneurons conditional knock-in mice (Sharma et al., 2020), indicating that hippocampal CA1 SOM interneurons are pivotal for memory consolidation. As mentioned above, these behavioral changes suggest that a reduction in eIF2α phosphorylation in SOM interneurons promotes memory formation *via* two mechanisms; first, it increases the responsiveness of PCs to SC inputs by disinhibition and thereby facilitates LTP at these synapses; and second, it suppresses LTP in the TA pathway, thereby modulating sensory inputs from the entorhinal cortex (Sharma et al., 2020). These findings suggest the existence of two autonomous and complementary memory consolidation processes mediated by eIF2α-dependent translational control in PCs and SOM interneurons: (i) translational changes in excitatory PCs help to facilitate memory consolidation by mediating synaptic plasticity in a sparse population of CA1 PCs; and (ii) translational changes in SOM interneurons facilitate memory consolidation by gating synaptic plasticity in the CA1 PCs circuit.

### Dorso-Ventral Differences in SOM Interneuron Function

The research reviewed so far on CA1 SOM interneurons and hippocampal function has been mostly concerned with interneurons of the dorsal hippocampus. However, recent work suggests differences in the function of CA1 SOM interneurons along the dorsoventral hippocampal axis (Siwani et al., 2018). Targeted expression of optogenetic tools in CA1 OLM cells expressing the nicotinic receptor α2 subunit (OLMα2) was used to activate or silence these cells in freely moving mice during contextual passive avoidance tasks and novel object recognition (Siwani et al., 2018). Activation of intermediate CA1 OLMα2 interneurons during passive avoidance learning impairs aversive memory, whereas silencing of OLMα2 cells has no effect. In contrast, silencing of dorsal CA1 OLMα2 interneurons impairs aversive memory (Siwani et al., 2018). For object recognition, silencing of intermediate CA1 OLMα2 interneurons during training enhances object memory, while their activation impairs it. In contrast, silencing dorsal CA1 OLMα2 cells has no effect on object memory (Siwani et al., 2018). To summarize, in contrast to dorsal CA1, intermediate CA1 OLMα2 cell activity is not required for contextual fear memory. However, their activation reduces both contextual fear and object memory (Siwani et al., 2018). Thus, intermediate OLMα2 cells can modulate object or fear-related representations. These findings suggest that intermediate OLMα2 cells may be silenced during fear memory formation, meaning that in the intermediate CA1, the inputs from the TAP to CA1 PCs may not be dampened in the learning process. This is consistent with the memory impairment induced by the optogenetic activation of these cells during the aversive stimuli presentation or during object exploration (Siwani et al., 2018). Alternatively, the memory impairment induced by intermediate OLMα2 cell activation may mean that proper memory formation requires the activation of a sparse number of cells and that optogenetic stimulation activates many of them. Interestingly, intermediate OLMα2 cells display increased sensitivity for acetylcholine compared to dorsal CA1 OLMα2 cells which could mean that these cells are more entrained by septal inputs and play a role in timing the inputs onto CA1 PCs. Thus, activating intermediate OLMα2 cells with optogenetics could have disrupted the proper convergence of inputs. Lastly, the increase of object recognition following intermediate OLMα2 cell inhibition during object exploration could indicate that their role is to gate the size of the engram encoding object memory, as seen in other hippocampal areas (see below, Stefanelli et al., 2016), and inhibiting these cells would lead to a stronger but less precise memory.

The role of SOM interneurons in contextual fear memory also appears different in the dentate gyrus (DG) than in the dorsal CA1 hippocampus (Stefanelli et al., 2016). Silencing DG SOM interneurons with inhibitory DREADD, during contextual fear conditioning, increases contextual fear memory and the size of the c-Fos expressing granule cell engram (Stefanelli et al., 2016). Activation of DG SOM interneurons using excitatory DREADD during contextual fear training, impairs contextual fear memory and reduces the number of c-Fos expressing granule cells (Stefanelli et al., 2016). Thus, DG SOM interneurons, most likely HIPP cells, may gate the size of the DG neuronal ensemble encoding contextual memory *via* dendritic lateral inhibition of granule cells (Stefanelli et al., 2016).

### SOM Interneurons and Place Cells

During spatial exploration, a subset of dorsal CA1 pyramidal cells progressively displays increased firing when the animal approaches a specific location. Each place cell demonstrates a preference for a different location (place field; O’Keefe and Dostrovsky, 1971). Place cells are characterized by a slow ramp-like depolarization of membrane potential (*Vm*) driving increased AP discharge when the animal passes through the place field (Harvey et al., 2009; Lee et al., 2012; Bittner et al., 2015). The fluctuations in PCs *Vm* drive their output firing, and the formation of place cells is mediated by synaptic potentiation of a specific subset of excitatory inputs (Bittner et al., 2015; Sheffield et al., 2017). In CA1 PCs, dendritic plateau potentials are generated by the coincident activation of CA3 and entorhinal cortex inputs, leading to increased output firing and LTP of perforant path synapses (Takahashi and Magee, 2009), and the induction of place field in PCs (Bittner et al., 2015).

Inhibitory interneurons play a cardinal role in achieving input selectivity necessary to drive place cells output firing (Grienberger et al., 2017). Optogenetic silencing of CA1 SOM or PV interneurons, or both populations, in awake head-fixed mice, performing a spatial navigation task increases PCs output firing in their place fields, without affecting their firing rate out-of-field (Royer et al., 2012; Grienberger et al., 2017). This is consistent with the dendrite-targeting interneurons role in regulating complex spiking and perisomatic-targeting interneurons regulating AP timing (Royer et al., 2012). Thus, inhibitory interneurons may be critical during place cell firing by controlling their excitatory inputs, by limiting dendritic amplification and suppressing out-of-field excitatory inputs (Grienberger et al., 2017). By that mean, SOM interneurons participate in the control of the plasticity of specific relevant inputs (Takahashi and Magee, 2009).

Recent works *in vivo* revealed that CA1 PCs exhibit place field plasticity following the initiation of dendritic plateau potentials occurring naturally in behaving mice or induced artificially by the injection of a depolarizing current through the recording pipette (Bittner et al., 2015, 2017). This synaptic plasticity is termed behavioral timescale synaptic plasticity (BTSP), in which active excitatory inputs within seconds before or after the generation of dendritic plateau potentials are selectively potentiated, contrary to the classical Hebbian plasticity in which a coincident activation of the presynaptic and the postsynaptic neuron within a narrow time window is required (Bittner et al., 2017; Magee and Grienberger, 2020). BTSP can be induced *ex vivo* in hippocampal acute slices by pairing stimulations of the presynaptic input with dendritic plateau potential in the postsynaptic PC, is pathway-specific, and requires the activation of NMDAR and L-type Ca^2+^ channels (Bittner et al., 2017; Magee and Grienberger, 2020). Interestingly, during spatial navigation, when an animal transits from familiar to a new environment, SOM interneurons-mediated dendritic inhibition transiently decreases which causes a short–lasting increase in PCs dendritic excitability, whereas during the same period, PV interneurons-mediated perisomatic inhibition increases (Sheffield et al., 2017; Sheffield and Dombeck, 2019). Notably, the transient decrease in dendritic inhibition may serve as a time window in which increased dendritic plateau potentials in PCs promotes synaptic potentiation to occur in selective inputs to drive place output firing once the inhibitory system recovers in the familiar environment.

Hence, SOM interneurons have a determining role in place field formation by regulating dendritic plateau potentials and synaptic plasticity induction in selective excitatory inputs necessary for network adaptation to environmental changes.

## SOM Interneurons and Astrocytes

In addition to pre- and post-synaptic neurons, glial cells and particularly astrocytes can actively modulate synaptic transmission in hippocampal circuits through bidirectional communication with neurons. The term “tripartite synapse” (Araque et al., 1999) encompasses the structural enwrapping of the synaptic cleft that allows astrocytes to sense neuronal activity through membrane receptors, leading to spatiotemporally coordinated fluctuations of intracellular Ca^2+^ levels and their ability to trigger gliotransmitters release (Araque et al., 2014; Bazargani and Attwell, 2016; Durkee and Araque, 2019). Despite multiple interactions between inhibitory networks and excitatory circuits (Klausberger and Somogyi, 2008), the current understanding of the bidirectional communications between neurons and astrocytes has emerged mainly from studies focusing on excitatory transmission leaving their involvement at inhibitory synapses ill-defined (Losi et al., 2014). However recent studies indicate that astrocytes also interact dynamically with inhibitory interneurons.

**Figure 6:**
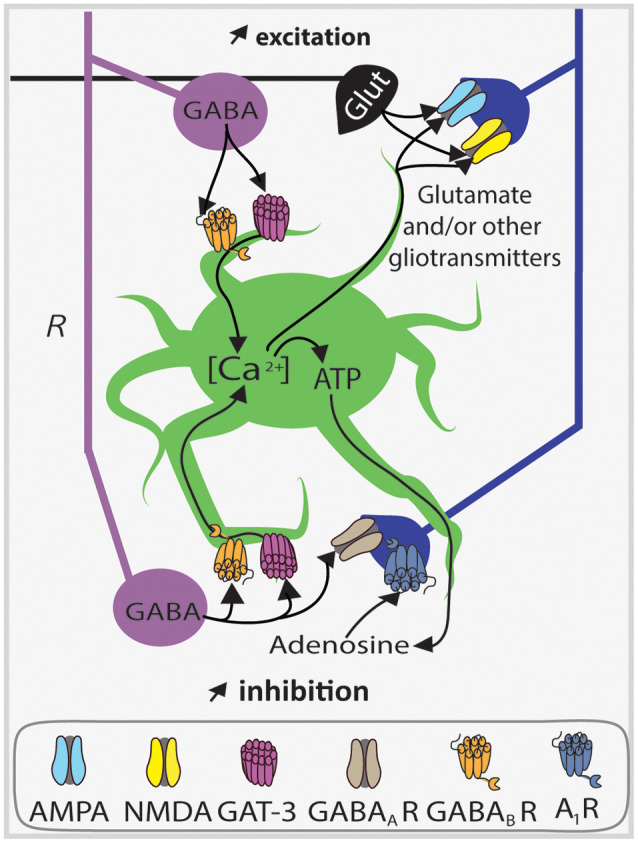
Astrocytes upregulate synaptic inhibition and excitation of PC apical dendrites via SOM interneuron GABA release. Diagram of endogenous astrocyte-mediated positive feedback autoregulation of SOM interneuron (violet) dendritic inhibition of CA1 PCs (blue) and putative regulation of excitatory inputs. Astrocytes (green) sense GABA release through the GABA transporter GAT-3 and GABA_**B**_Rs which induce an increase in astrocyte calcium concentration ([Ca^2+^]) leading to the liberation of ATP in the extracellular compartment. After its release, ATP is converted into adenosine, which then activates pyramidal cell adenosine 1 receptors (A_1_Rs). Activation of these receptors enhances SOM interneuron synaptic inhibition by a mechanism leading to gain of function of postsynaptic GABA_A_Rs. Parallel to this mechanism, as a consequence of Ca^**2+**^ rise, astrocytes can probably release other gliotransmitters like glutamate to coordinate different synapses (black, excitatory synapse). Abbreviations: Glut, Glutamate; R, *radiatum*.

Through expression of GABA receptors [GABA_A_Rs (Egawa et al., 2013; Ishibashi et al., 2019) and GABA_B_Rs (Kang et al., 1998; Serrano et al., 2006; Ding et al., 2009; Haustein et al., 2014; Ishibashi et al., 2019)] and transporters (GAT-1 and GAT-3; Borden and Caplan, 1996; Ribak et al., 1996; Ishibashi et al., 2019), astrocytes can detect and respond to GABAergic activity with Ca^2+^ oscillations (Nilsson et al., 1993; Lia et al., 2019). Astrocytic GABAergic Ca^2+^ activities are mediated by several mechanisms involving voltage-sensitive Ca^2+^ channels, release from internal stores, G proteins, GATs, and sodium/calcium exchangers (NCXs; Perea et al., 2016; Ishibashi et al., 2019; Mederos and Perea, 2019; Lia et al., 2019). GABAergic activation of astrocytes can lead to the release of various gliotransmitters such as glutamate (Kang et al., 1998; Andersson et al., 2007; Mariotti et al., 2016; Perea et al., 2016; Mederos and Perea, 2019), GABA (Lee et al., 2010; Yoon et al., 2011), ATP (Serrano et al., 2006; Boddum et al., 2016; Covelo and Araque, 2018; Matos et al., 2018) or efflux of chloride (Egawa et al., 2013) to modulate both excitatory and inhibitory transmission (Figure 6; Perea et al., 2016). While the heterogeneity of interneurons subtypes can give rise to diverse GABAergic signaling, we focus here on the interplay between hippocampal SOM interneurons, PCs, and astrocytes.

Intense depolarization of astrocytes induces Ca^2+^ increases that potentiate miniature inhibitory postsynaptic currents (mIPSCs) in pyramidal cells (Kang et al., 1998), that could originate at least in part from SOM interneurons with axons in *stratum radiatum* (e.g., BiC). In addition, calcium uncaging in hippocampal astrocytes leads to an increase in spontaneous inhibitory postsynaptic currents (sIPSCs) frequency in *stratum radiatum* interneurons, which is induced by astrocytic glutamate release targeting kainate receptors on PCs (Liu et al., 2004). These studies show that astrocytes are well suited to establish another level of connection and regulation between inhibitory and excitatory networks in the hippocampus. Indeed GABAergic activation of hippocampal astrocytes was found to induce the release of ATP, which is converted extracellularly into adenosine in order to activate presynaptic adenosine A_1_ receptors (A_1_R) on PCs in the context of heterosynaptic depression at excitatory synapses of CA1 PCs (Serrano et al., 2006). This demonstrated a functional interplay between glial and GABAergic neuronal networks during heterosynaptic plasticity.

More recently, the use of optogenetic tools (Goshen, 2014) allowed the specific stimulation of different subtypes of interneurons to decipher the complex modulatory mechanisms of inhibitory synapses. In acute hippocampal slices, astrocytes specifically upregulate synaptic inhibition of PCs by SOM interneurons but not PV interneurons (Matos et al., 2018). Using the cell-specific expression of channelrhodopsin-2 in SOM or PV interneurons, optogenetic activation of SOM interneurons (evoking one or two action potentials) was found to induce Ca^2+^ activities in astrocyte processes mediated by GABA_B_R and GAT-3 (Figure 6; Matos et al., 2018). This in turn leads to the astrocytic release of ATP, converted extracellularly into adenosine that acts on postsynaptic A_1_R to upregulate SOM interneuron inhibition onto PCs (Figure 6). This suggests an endogenous astrocyte-mediated positive feedback autoregulation of SOM interneuron dendritic inhibition of PCs (Matos et al., 2018). Importantly, this mechanism may be specific to SOM interneuron synapses as blockers of A_1_R (DPCPX) or GAT-3 (SNAP-5114) failed to affect IPSCs evoked in PCs by optogenetic activation of PV interneurons (Matos et al., 2018). In addition, spontaneous synaptic inhibition (sIPSC) in PCs (which reflects more global inhibition of PCs coming from many interneurons subtypes), was depressed by inhibition of A_1_R, GAT-3, or astrocytic Ca^2+^ oscillations (Matos et al., 2018). These findings point toward the possible existence of multiple astrocyte-mediated modulations of synaptic inhibition originating from different interneuron subtypes.

Consistent with this concept, other groups have shown different astrocyte modulation based on interactions with specific interneuron subtypes. Optogenetic activation of CA1 hippocampal astrocytes in slices increases the firing rate of CCK interneurons, but not PV interneurons, dependent on the release of ATP acting on P2Y-1R which leads to inhibition of a two-pore domain potassium channel (K2P; Tan et al., 2017). In contrast, optogenetic activation of astrocytic ATP release induces hyperpolarization of PCs mediated by its conversion into adenosine acting on A_1_R (Tan et al., 2017). Interestingly in the neocortex, astrocyte Ca^2+^ elevations are differentially modulated by GABAergic signaling originating from PV or SOM interneurons. Optogenetic activation of PV interneurons induces weak Ca^2+^ elevations whereas SOM interneuron activation results in robust GABA_B_ receptor-mediated Ca^2+^ elevations (Mariotti et al., 2018). Moreover, these astrocyte Ca^2+^ responses present a form of plasticity, with depression of Ca^2+^ elevations upon repetitive PV interneuron stimulations and enhancement after repetitive SOM interneuron stimulations. The latter relies on somatostatin released by SOM interneurons acting on somatostatin receptors along with astrocytic processes (Mariotti et al., 2018).

Taken together these results show that astrocytes dynamically influence both the input and output of inhibitory interneurons, notably SOM interneurons, and thus, astrocytes do not have only a passive role in inhibitory circuits, similarly to their contribution to excitatory circuits. Astrocytes are at the interface between excitatory and inhibitory synapses, and more detailed and comprehensive studies *in situ* and *in vivo* are required to refine our understanding of the inhibitory-glial-excitatory networks interplay involving SOM interneurons and its role in hippocampal synaptic plasticity and function.

## SOM Interneurons in Disease

Given that hippocampal synaptic function and plasticity are impaired in brain disease with cognitive disorder, notably Alzheimer’s disease (AD), SOM interneuron dysfunction may also contribute to these cognitive impairments.

In his initial observations after the death of the patient A. Deter suffering from dementia, Alois Alzheimer identified two characteristic cerebral lesions, neurofibrillary tangles made of Tau and senile plaques consisting of Aβ peptide aggregation (Graeber et al., 1997). Accumulation of Aβ_1–42_ oligomers is one of the earliest events leading to direct or indirect synaptic alterations in AD (Huang and Mucke, 2012; Mucke and Selkoe, 2012). Electron microscopy and immunohistochemistry quantifications reported a significant decrease of synaptic density in the hippocampus of AD patients (Davies et al., 1987; Masliah et al., 2001). There is also a strong correlation between Aβ load in the patient brain and the extent of synapse loss (Wang et al., 1999). However, in many animal models of AD, despite cognitive alterations, these dendritic spine losses are not always present or as pronounced as in the human form of the disease (Auld et al., 2002; Elder et al., 2010). Accordingly, previous works have shown that different neuronal populations such as excitatory neurons vs. inhibitory interneurons, are differentially affected (Davies et al., 1980; Ramos et al., 2006). Moreover, inhibitory interneuron damage may occur before principal neuron alterations and the manifestation of symptoms. Focusing on SOM interneurons, a selective and early neurodegeneration of O-LM and HIPP cells was reported (Ramos et al., 2006). In the APP/PS1 transgenic mouse model of AD, several features of these SOM interneurons are altered. Using quantitative RT-PCR, SOM mRNA level is decreased as early as 4 months of age. Quantification of SOM interneurons showed a 60% reduction in cell numbers and the presence of dystrophic SOM interneurons in transgenic compared to wildtype animals (Ramos et al., 2006). Importantly, these alterations precede PC loss. Moreover, SOM interneuron modifications showed a linear relationship with Aβ presence/concentration indicating that SOM interneuron loss is an early hippocampal neuropathology in this mouse model of AD.

Functional alterations of hippocampal SOM interneurons also accompany the early neuropathological changes in the AD mouse model. In an elegant *in vivo* study taking advantage of Gad1-eGFP mice crossbred with APP/PS1AD mouse model, structural changes consisting of SOM interneuron axon losses were observed at 4 months of age (Schmid et al., 2016). Moreover, chronic imaging of individual eGFP positive O-LM cells revealed that the normal age-dependent increase in the number of SOM interneuron dendritic spines is impaired in APP/PS1 mice. As for excitatory neurons (Koffie et al., 2009; Wei et al., 2010), the reduction in SOM interneuron spine density was correlated with Aβ proximity (<50 μm). Nevertheless, spine stability was impaired in APP/PS1 mice with a greater spine turnover associated with Aβ distance. These structural modifications may represent primary mechanisms to cope with pathological alterations of synapses during AD (Schmid et al., 2016). However, contextual fear learning-associated plasticity of dendritic spines of SOM interneurons is also affected in APP/PS1 mice. *In vivo* imaging showed that, after contextual fear conditioning, the gain of SOM interneuron dendritic spines is impaired in APP/PS1 mice, and this is associated with impaired contextual fear memory, compared to control mice (Schmid et al., 2016). Based on the cholinergic input that O-LM cells receive from the medial septum during aversive stimuli (Lovett-Barron et al., 2014) and the well-documented cholinergic degeneration in AD (Auld et al., 2002; Perez et al., 2007), the reduction in spine formation could be an indirect result of presynaptic cholinergic deficits. Using imaging with GCaMP6m calcium indicator, delivery of aversive air puffs in awake head-fixed mice evoked reduced Ca^2+^ responses in putative O-LM cells of APP/PS1 mice (Schmid et al., 2016). These observations are consistent with the observed reduction of medial septum trans-synaptically labeled monosynaptic afferents to SOM interneurons in APP/PS1 mice. In addition, pharmacological blockade of m1AChR or chemogenetic silencing of SOM interneurons during fear conditioning was sufficient to mimic the spine gain reduction and impair fear memory in control mice. Conversely, application of the m1AChR agonist Cevimeline during fear conditioning restored fear memory in APP/PS1 mice (Schmid et al., 2016). These findings demonstrate that early alterations in SOM interneuron spine plasticity may be linked to behavioral impairment and memory loss in AD. Thus, targeting presynaptic inputs or postsynaptic SOM interneurons may offer complementary therapeutic strategies. Indeed, strategies targeting somatostatin receptor subtype-4 (SST_4_R) with agonists have recently been proposed to promote and restore the expression of altered subcortical mRNA genes in AD (Sandoval et al., 2019).

Another key function of SOM interneurons is their involvement in the generation of theta rhythms, which among other hippocampal network oscillations are impaired in AD models (Villette et al., 2010; Palop and Mucke, 2016; Mondragon-Rodriguez et al., 2018). An alternative to transgenic AD models is to inject directly soluble oligomers of Aβ peptide (Aβo). Recently, co-injection of Aβo and AAV5-Ef1a-DIO-hChR2(ET/TC)-mCherry to SST-Cre mice hippocampi was shown to replicate theta oscillations impairments (Villette et al., 2010; Palop and Mucke, 2016), and optogenetic activation of SOM interneurons restored the power of theta oscillations in Aβo injected animals (Chung et al., 2020). In addition, Aβo injection desynchronized SOM interneuron firing relative to theta oscillations, which was restored by optogenetic activation of SOM interneurons (Chung et al., 2020). Finally, in slices from Aβo-injected animals, optogenetic stimulation of SOM interneurons enhances sIPSCs received by CA1 PCs at theta frequencies (Chung et al., 2020). Together with the reported loss of SOM interneurons in the perirhinal cortex of AD patients correlated with Aβ load (Sanchez-Mejias et al., 2020), these findings suggest that targeting SOM interneurons may help restore network oscillations, which are heavily impacted during AD (Palop and Mucke, 2016). Interestingly, transplantation of interneuron progenitors can restore learning and memory in APOE4-KI mice in the presence of Aβ (Tong et al., 2014). Thus, maintaining proper SOM interneurons synaptic function and plasticity could be crucial to reduce the impact of AD on cognitive functions, as soon as Aβ production starts, or even later.

## Conclusions and Future Directions

The rapid pace of development of new experimental tools for cell-specific identification and manipulation of distinct neuron types will likely provide the means to address many interesting questions that remain unresolved about long-term plasticity at excitatory synapses of SOM interneurons and its role in hippocampal memory processes.

As mentioned above, hippocampal SOM interneurons are part of a group composed of many different cell types, even in a specific hippocampal region like CA1 (Pelkey et al., 2017). Presently, studies on synaptic plasticity of SOM interneurons have focused largely on CA1 BiC and OLM cells (Perez et al., 2001; Lamsa et al., 2007). However, other types of SOM interneurons include cells with both local and long-range projections (DP, BP, and ORP cells; Gulyas et al., 2003; Goldin et al., 2007; Jinno et al., 2007). The DP cells that project to the septum, as well as other hippocampal areas, may be particularly interesting to investigate given the crucial role of septal afferents to the hippocampus during spatial and contextual learning (Lovett-Barron et al., 2014; Schmid et al., 2016). Coupling of trans-synaptic monosynaptic labeling with DP cell-specific identification could be used to characterize and manipulate their excitatory synapses and potential long-term plasticity. It would be of interest to determine how the plasticity of DP interneuron excitatory synapses may play a role in coordinating changes across septal and hippocampal areas during hippocampal learning.

Another interesting issue is the regional difference in the function of SOM interneurons in hippocampal learning, and particularly that of OLM cells along the CA1 dorsoventral axis (Lovett-Barron et al., 2014; Siwani et al., 2018). The different roles of OLM cells in hippocampal learning were suggested to be due to a difference in septal cholinergic inputs along the dorsoventral axis (Siwani et al., 2018). As long-term plasticity of SOM interneuron synapses in hippocampal learning has been examined mostly in the dorsal hippocampus (Artinian et al., 2019), it would be of interest to examine if there is a difference in SOM interneuron long-term synaptic plasticity along the dorsal-ventral axis, and if so, does it parallel the difference in the functional role of SOM interneurons in hippocampal learning.

Much progress has been achieved on characterizing Hebbian and mGluR1-mediated LTP at excitatory synapses of SOM interneurons, and its role in the regulation of hippocampal network and memory (Artinian et al., 2019). However other types of long-term synaptic plasticity occur at excitatory synapses of SOM interneurons, notably anti-Hebbian LTP at BiC and OLM cell synapses (Lamsa et al., 2007), LTP at HIL cell synapses, and LTD at HIPP cell synapses (Yuan et al., 2017). These multiple types of synaptic plasticity may each supports a different function in the formation and consolidation of hippocampus-dependent memory. Therefore, it would be important to demonstrate these roles in hippocampal function using cell-specific manipulations and hippocampal learning tasks.

Another largely unresolved question is the role of the release of the endogenous peptide SOM in SOM interneuron function. Ablation of the SOM gene or depletion of SOM by cysteamine treatment impairs LTP in CA1 PCs and impedes contextual fear memory (Kluge et al., 2008). Similarly, blocking LTP at excitatory synapses of SOM interneurons decreases contextual fear memory and prevents the facilitation of SC-PC LTP by SOM interneuron synaptic plasticity (Artinian et al., 2019). These effects on contextual fear memory and on CA1 PC synaptic plasticity suggest a possible link between the peptide SOM and long-term plasticity at SOM interneuron excitatory synapses. This would be interesting to explore given that endogenous SOM release is considered to be activity-dependent.

Work on astrocyte regulation of SOM interneuron synapses focused largely on astrocyte interactions at inhibitory synapses made by SOM interneurons on PC dendrites (Matos et al., 2018). However, astrocyte interactions are largely documented at excitatory synapses onto PCs and linked to regulation of long-term plasticity at these synapses (Araque et al., 2014). Interestingly, the activity of astrocytic glutamate transporters GLT-1 and GLAST regulate mGluR1-mediated slow EPSCs in CA1 OLM cells, indicating a functional interaction of astrocytes at excitatory synapses onto SOM interneurons (Huang et al., 2004). Thus, it would be interesting to characterize further astrocyte interactions at these excitatory synapses and determine how astrocytes may influence long-term synaptic plasticity of SOM interneurons, and consequently, hippocampal learning and memory.

Astrocytes also display functional heterogeneity, at least in terms of GABAergic-induced Ca^2+^ oscillation and gliotransmitter release. A single astrocyte can be modulated by distinct mechanisms (endocannabinoids and GABA) and can release at least two different gliotransmitters (ATP/adenosine and glutamate; Covelo and Araque, 2018). Given the different astrocytic modulation uncovered with SOM and other interneuron subtypes (Mariotti et al., 2018; Matos et al., 2018), it raises the question whether different subtypes of astrocytes may co-exist within a brain region. Astrocyte heterogeneity has already been reported between brain regions (Chai et al., 2017; Khakh and Deneen, 2019; Kohler et al., 2021). However, given the different firing properties of specific interneurons, do specific firing patterns govern the astrocytic responses or is it another level of modulation? These questions still need to be addressed.

Future progress on these and other questions will likely move forward our understanding of SOM interneuron synaptic plasticity and help uncover how these specific inhibitory cells contribute to hippocampal memory processes.

## Author Contributions

All authors contributed to the article and approved the submitted version.

## Conflict of Interest

The authors declare that the research was conducted in the absence of any commercial or financial relationships that could be construed as a potential conflict of interest.
